# Biginelli Reaction Mediated Synthesis of Antimicrobial Pyrimidine Derivatives and Their Therapeutic Properties

**DOI:** 10.3390/molecules26196022

**Published:** 2021-10-04

**Authors:** Maria Marinescu

**Affiliations:** Department of Organic Chemistry, Biochemistry and Catalysis, Faculty of Chemistry, University of Bucharest, Soseaua Panduri, 030018 Bucharest, Romania; maria.marinescu@chimie.unibuc.ro

**Keywords:** Biginelli reaction, dihydropyrimidininones, catalyst, antimicrobials, antitubercular, antioxidant

## Abstract

Antimicrobial resistance was one of the top priorities for global public health before the start of the 2019 coronavirus pandemic (COVID-19). Moreover, in this changing medical landscape due to COVID-19, finding new organic structures with antimicrobial and antiviral properties is a priority in current research. The Biginelli synthesis that mediates the production of pyrimidine compounds has been intensively studied in recent decades, especially due to the therapeutic properties of the resulting compounds, such as calcium channel blockers, anticancer, antiviral, antimicrobial, anti-inflammatory or antioxidant compounds. In this review we aim to review the Biginelli syntheses reported recently in the literature that mediates the synthesis of antimicrobial compounds, the spectrum of their medicinal properties, and the structure–activity relationship in the studied compounds.

## 1. Introduction

**Scheme 1:**
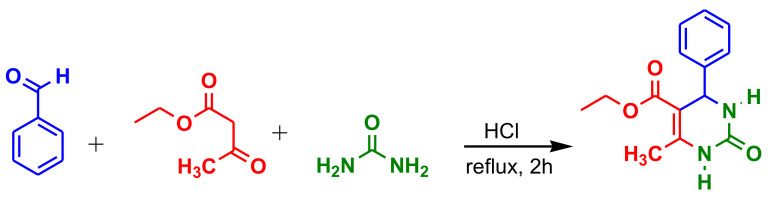
The Biginelli dihydropyrimidone synthesis.

Twelve years after 1881, the year in which the German Arthur Rudolf Hantzsch reported the multicomponent synthesis of dihydropyridine [1], in 1893, the Italian chemist Pietro Biginelli published the synthesis of 3,4-dihydropyrimidin-2(1*H*)-ones, by a simple one-pot condensation reaction of an aromatic aldehyde, urea, and ethyl acetoacetate in ethanol solution (Scheme 1) [2,3]. Both reactions proved to be “key methods” for the synthesis of pyridine and pyrimidine derivatives, respectively, which were greatly developed in the following period, especially due to the applications of the synthesized compounds. The Biginelli reaction has been intensively studied in the last two decades, especially due to the applications of synthesized dihydropyrimidinone compounds at the beginning, especially as calcium channel blockers of the nifedipine-type [4], and then as antitumor [5,6,7,8], antibacterial, antiviral [9,10], anti-inflammatory [11,12], analgesic [13], anti-Alzheimer [14], or antioxidant [15] compounds.

The synthetic method initially reported by Biginelli has undergone changes, such as the “Atwal-modification” [4,5], and most often, the most efficient catalyst has been sought, which would lead to a higher product yield, milder reaction conditions, and efficient catalyst recovery [16,17]. In the last decade, several improved methods were reported for the Biginelli synthesis of these compounds, including solvent free synthesis [18], ultrasound radiation [19], microwave irradiation [20], visible light irradiation [21], or using a biocatalyst [22]. Further methods include using various catalysts, such as Bronsted acids, including H_3_BO_3_ [15], HCOOH [21], p-TsOH-H_2_O [23,24], imidazole-1–yl-acetic acid [25], and L-(+)-tartaric acid-dimethylurea [26]; or Lewis acids, including LiClO_4_, Lal_3_, InCl_3_, BiCl_3_, Bi(OTf)_3_, Mn(OAc)_3_, Cu(OTf)_2_, CuCl_2_, FeCl_3_, ZrCl_4_, SnCl_2_, [27,28,29,30,31,32], Sr(OTf)_2_ [33], VCl_3_ [34], TaBr_5_ [35], Ce(NO_3_)_3_·6H_2_O [36], ZrO_2_/SO_4_ ^2−^ [37], silica-chloride (SiO_2_-Cl) [38], Sm(ClO_4_)_3_ [39], Y(NO_3_)_3_·6H_2_O [40], CeCl_3·_7H_2_O [41], Ce(NH_4_)_2_(NO_3_)_6_ (CAN) [42], Fe(OTs)_3·_6H_2_O [43], Ca(HSO_4_)_2_, Zn(HSO_4_)_2_ [44], SnCl_2_/nano SiO_2_ [45], Cu(OAc)_2_ [46], copper zirconium phosphate Cu(OH)_2_Zr(HPO_4_)_2_ [47], Sc(OTf)_3_, Yb(OTf)_3_, and Zn(OTf)_2_ [48]. Other catalysts of the Biginelli reaction reported in the literature include co-phthalocyanines [49], NaHSO_4_ [50], zeolites [51,52], clays [53,54,55], organic polymers [56,57], organic–inorganic mesoporous materials [58], ionic liquids [59,60,61], etc. [62,63,64]. Asymmetric Biginelli syntheses have also been developed due to the special importance of the pharmaceutical properties of optically active dihydropyrimidinone compounds [65,66,67]. For Biginelli synthesis, the literature accepts three plausible mechanisms: through the formation of the imine intermediate, through the formation of the enamine intermediate, and through the formation of the Knoevanagel intermediate [66]. Despite this, it can be assumed that the Biginelli reaction is, in fact, a multicomponent reaction catalyzed by urea through the generation of iminium species, energetically favored route [32,66].

**Figure 1:**
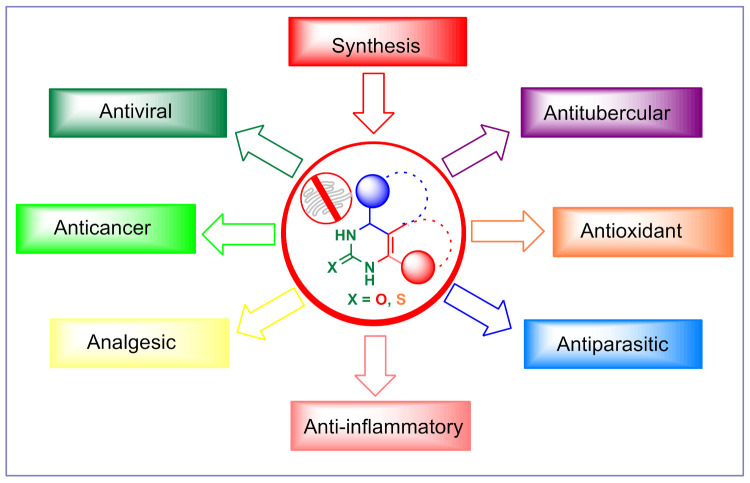
Schematic representation of the synthesis and therapeutical properties of antimicrobial Biginelli mediated Pyrimidine compounds.

The need to find new compounds with remarkable antimicrobial activities and with a wide range of other therapeutic activities has been stimulated in the last two years by the fact that both patients with severe cases and patients with moderate cases of COVID-19, with or without pneumonia, they received treatments with various antibiotics [68,69]. Therefore, given the various dihydropyrimidinone compounds synthesized through the Biginelli reaction, as well as their various biological properties (Figure 1), in the present study, we aim to present the Biginelli syntheses that led to compounds with antimicrobial properties as well as their therapeutic properties. The database search methodology used in this review was the use of keywords, which can be found in the title, such as Biginelli reaction, pyrimidine derivatives, and Biginelli therapeutic properties, in different websites, such as PubMed, MDPI, Science Direct, Springer, The Royal Society Chemistry, ACS Publications, and Taylor & Francis. The selection of scientific articles for the last ten years was made according to the novelty brought in the Biginelli synthesis, the subsequent synthesis scheme, as well as the therapeutic properties of the reported compounds. In general, articles from the last ten years have been selected.

## 2. Biginelli Reaction Mediated Synthesis of Antimicrobial Pyrimidine Derivatives

**Scheme 2:**
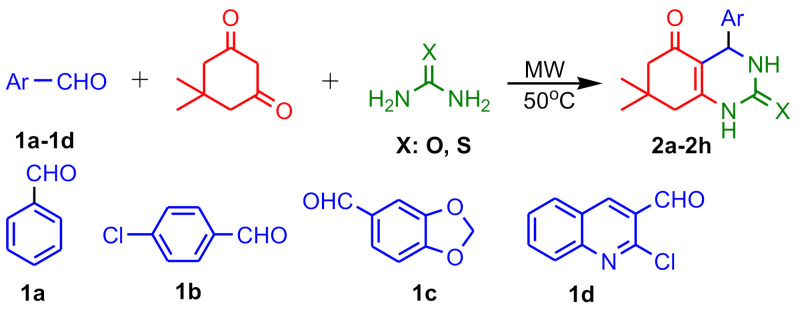
Synthesis of quinazolines **2a**–**2h** using Biginelli reaction.

Kidwai et al. developed a convenient synthetic route for the preparation of quinazolines **2a**–**2h** by heating equimolar amounts of aldehyde **1a**–**1d**, 5,5-dimethyl-1,3-cyclohexanedione (dimedone), and urea/thiourea in the absence of solvent and catalyst, under microwave irradiation (Scheme 2) [70]. All compounds **2a**–**2h** showed antibacterial activity against *Staphylococcus aureus* ATCC 25923, *Escherichia coli* ATCC 25922 and *Pseudomonas aeruginosa* ATCC 27853 in the concentration range of 0.564 μg mL^−1^ when tested by broth microdilution MIC method using norfloxacin as the standard drug.

**Scheme 3:**
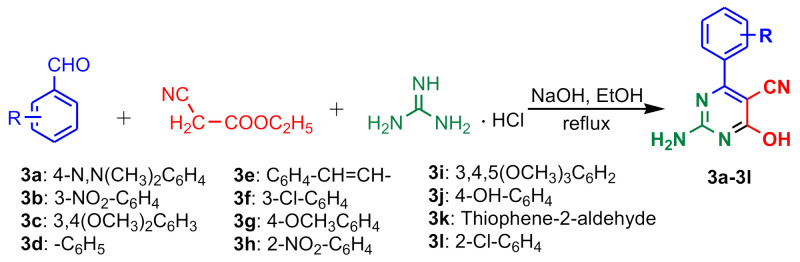
Synthesis of 2-amino-5-cyano-6-hydroxy-4-arylpyrimidines **3a**–**3l**.

Deshmukh et al. reported an efficient one step synthesis of new 2-amino-5-cyano-6-hydroxy-4-arylpyrimidines **3a**–**3l** by three component Biginelli condensation of aromatic aldehydes, ethyl cyanoacetate, and guanidine hydrochloride in alkaline ethanol (Scheme 3) [71]. All synthesized compounds showed good to excellent activity against tested Gram-positive (*S. aureus*) and Gram-negative (*E. coli*) bacteria, but 2-amino-4-hydroxy-6-phenylpyrimidine-5-carbonitrile **3d** was found to be selectively active against Gram-positive bacteria.

**Scheme 4:**
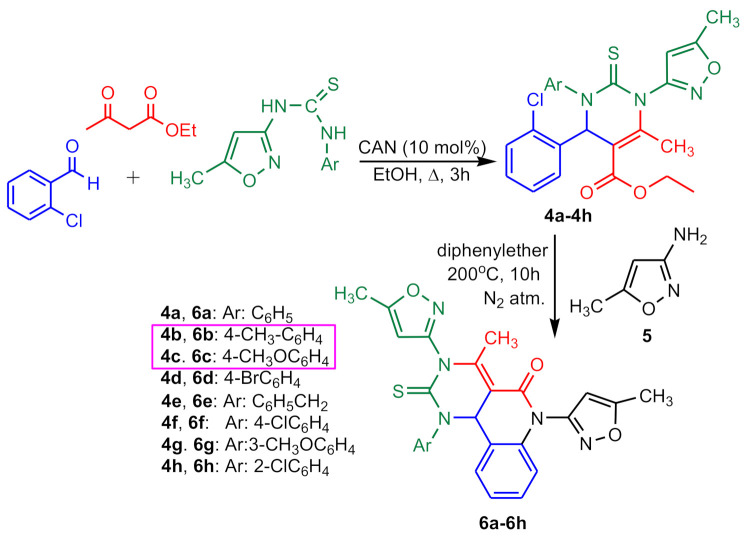
Synthesis of compounds **4a**–**4h** and **6a**–**6h**.

Rajanarendar et al. reported Biginelli one-pot condensation of 2-chlorobenzaldehyde, ethyl acetoacetate, and 1-(5-methylisoxazol-3-yl)-3-phenyl thioureas in presence of 10 mol% of ceric ammonium nitrite (CAN) in methanol at 80 °C for 3 h, to obtain isoxazolyl-dihydropyrimidine-thione carboxylates **4a**–**4h** in 80–90% yields. On heating compound **4** with 3-amino-5-methylisoxazole **5** for 10 h in diphenyl ether at 200 °C under nitrogen atmosphere, new cyclization occurred, yielding 2-thioxo-2,3,6,10*b*-tetrahydro-1*H*-pyrimido[5,4-c]quinolin-5-one compounds **6a**–**6h** (Scheme 4) [72]. Compounds **6a**–**6h** exhibited moderate to good antibacterial activity against *Bacillus subtilis* MTCC 441, *Bacillus sphaericus* MTCC 511, *Staphylococcus aureus* MTCC 96, *Pseudomonas aeruginosa* MTCC 741, *Klebsiella aerogenes* MTCC 39, and *Chromobacterium violaceum* MTCC 2656, and are more active than the standard drug Ciprofloxacin. The antifungal activity of compounds **6a**–**6h** showed that they are significantly toxic towards all the five tested pathogenic fungi, *Aspergillus niger* MTCC 282, *Chrysosporium tropicum* MTCC 2821, *Rhizopus oryzae* MTCC 262, *Fusarium moniliformae* MTCC 1848, and *Curvularia lunata* MTCC 2030, and they are lethal even at a 100 μg mL^−1^ concentration. However, compounds **6b** and **6c** exhibited high activity, and they inhibited the growth of fungi to a remarkable extent, which correlated with the presence of methyl and methoxy substituents on the *para* position of the benzene ring.

**Scheme 5:**
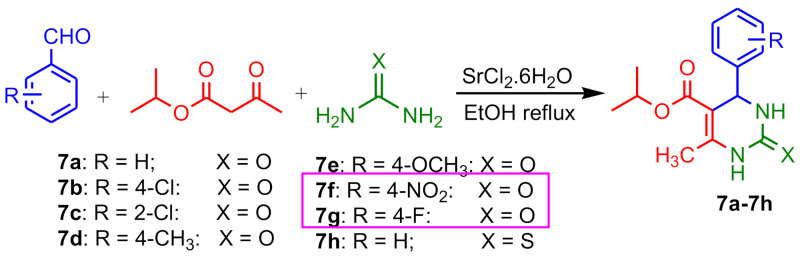
Synthesis of compounds **7a**–**7h**.

Chitra et al. reported the synthesis of Biginelli compounds **7a**–**7h** by a one pot cyclocondensation of aldehydes, isopropyl acetoacetate, and urea/thiourea in ethanol, using strontium chloride hexahydrate as the catalyst (Scheme 5) [73]. Generally, the compounds showed moderate-to-good antibacterial activity against *Staphylococcus aureus*, *Escherichia coli*, *Klebsiella pneumoniae*, *Pseudomonas aeruginosa*, and *Salmonella typhi*. Only **7f**, which has a *nitro* group at the *para* position, and **7g**, which has a *fluorine* group in the *para* position, are more active than the reference drug Ciprofloxacin. Additionally, the antifungal activity against *Candida albicans*, *Aspergillus flavus*, *Rhizopus*, and *Mucor* of compounds **7f** and **7g** are better than of the standard drug Amphotericin B against all the tested organisms. It is also noted that compounds **7b**–**7g**, which have substituents in the 4-aryl group, are more active than the parent compound **7a** against all the tested fungi.

**Scheme 6:**
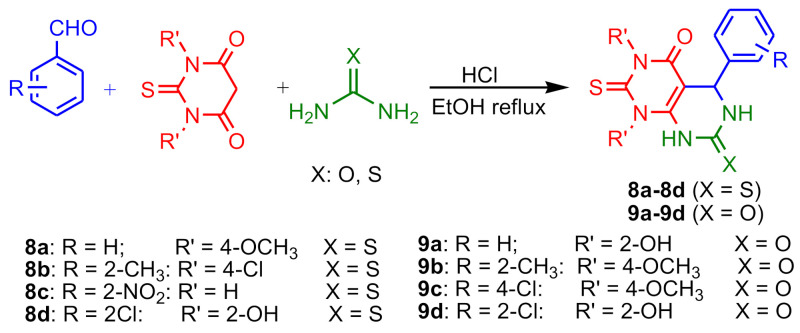
Synthesis of compounds **8a**–**8d** and **9a**–**9d**.

Dabholkar et al. reported the one-step synthesis of dihydropyrimidinones **8a**–**8d** and **9a**–**9d**, using a classic Biginelli synthesis, from thiobarbituric acid, aromatic aldehyde, urea/thiourea in ethanol, and a catalytic amount of HCl (Scheme 6) [74]. All synthesized compounds showed good antibacterial activity against *Staphyllococcus aureus* ATTC-27853, *Corynebacterium diphtheria* ATTC-11913, *Proteus aeruginosa* (recultured) and *Escherichia coli* ATTC-25922 bacterial strains by the disc diffusion method, considering Ampiciline trihydrate as the standard drug.

**Scheme 7:**
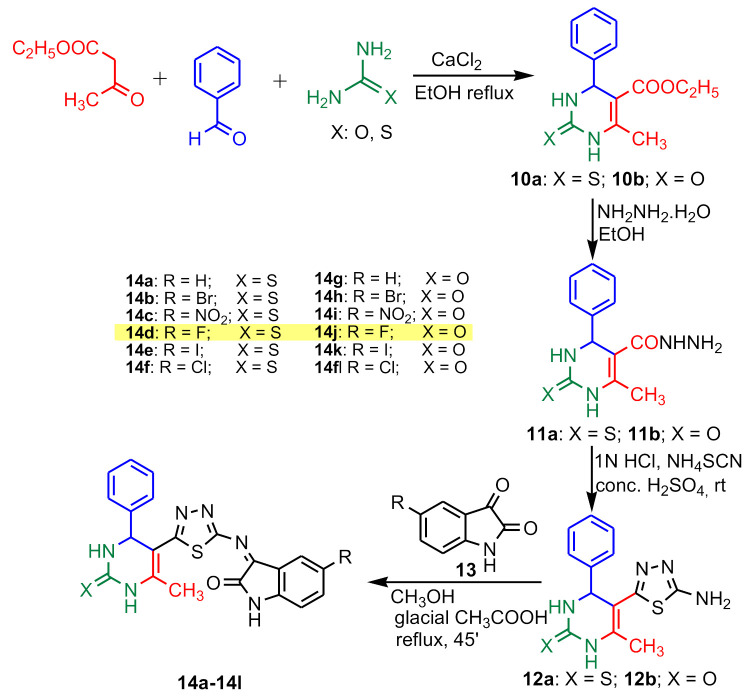
Synthesis of compounds **14a**–**14l**.

Akhaja et al. proposed a method for the synthesis of 1,3-dihydro-2H-indol-2-ones derivatives **14a**–**14l** in 4 steps: (1) Biginelli synthesis on CaCl_2_ catalyst (compounds **10**), (2) synthesis of hydrazides **11**, by treatment of Biginelli compounds with hydrazine hydrate, (3) cyclization to 1,3,4-thiadiazole **12** in concentrated H_2_SO_4_ medium, and (4) condensation with various 5-substituted indoline-2,3-dione **13**, in acidic medium, to afford the final compounds **14** (Scheme 7) [75]. Antibacterial activity of all synthesized compounds was screened against *Escherichia coli* MTCC-443, *Pseudomonas aeruginosa* MTCC-1688, Klebsiella pneumonia MTCC-109, *Salmonella typhi* MTCC-98, *Staphylococcus aureus* MTCC-96, Staphylococcus pyogenus MTCC-442, and *Bacillus subtilis* MTCC-441, with Gentamycin, Ampicillin, Chloramphenicol, Ciprofloxacin, and Norfloxacin used as standard antibacterial agents; additionally, antifungal activity was screened against three fungal species, *C. albicans* MTCC 227, *Aspergillus niger* MTCC 282, and *Aspergillus clavatus* MTCC 1323, with Nystatin and Griseofulvin as standard antifungal agents. It was found that compounds **14d** and **14j** (MIC = 62.5–100 μg mL^−1^), containing a strong electron withdrawing group (F), exhibit excellent activity against all bacterial strains, while **14b** and **14h** (with Br) exhibited comparable activity against Gram-positive strains (MIC = 100–250 μg mL^−1^), and **14c** and **14i** (with NO_2_) are highly active against Gram-negative strains (MIC = 100–250 μg mL^−1^), as compared to standard antibiotic Ampicillin. Additionally, compounds **14d** and **14f** possessed the highest antifungal activity against all fungal strains (100 μg mL^−1^). It was established that the order of decrease in antibacterial activity, depending on the substituent present at the 5th position of 1H-indole-2,3-diones, is F > NO_2_ > Br > Cl > H.

**Figure 2:**
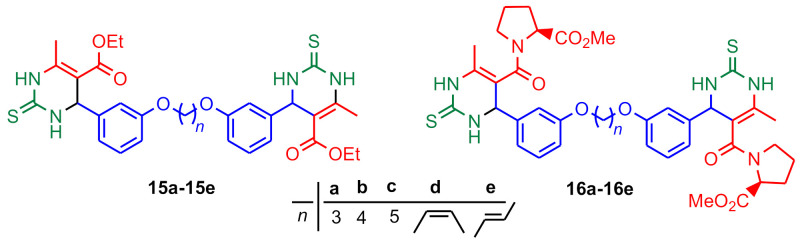
Chemical structures of the comounds **15a**–**15e** and **16a**–**16e**.

Kamal et al. reported the Biginelli synthesis of some conformationally flexible dimers of monastrol **15a**–**15e** and **16a**–**16e**, using CeCl_3_.7H_2_O as catalyst (Figure 2) [76]. Compounds **15a**, **15d**, **15e,** and **16d** exhibited moderate activity against Gram-positive bacteria, such as *Staphylococcus aureus*, *Staphylococcus epidermidis*, *Bacillus subtilis*, and Gram-negative bacteria, including *Escherichia coli*, *Klebsiella pneumoniae*, and *Pseudomonas aeruginosa*.

**Scheme 8:**
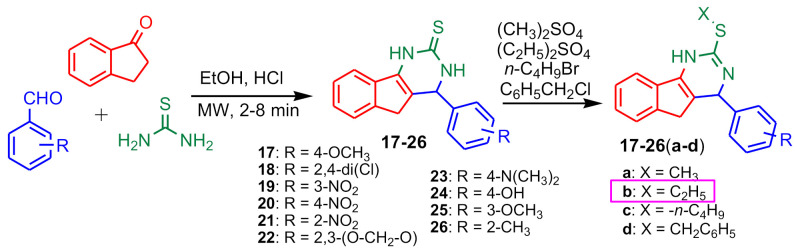
Synthesis of compounds **17**–**26** and **17**–**26**(**a**–**d**).

Kaur et al. reported the synthesis of alkylated indeno[1,2-d] pyrimidine-2-thiones **17**–**26** (**a**–**d**) in two steps: (i) Biginelli condensation under microwave irradiations to obtain compounds **17**–**26**, and (ii) alkylation of the 3,4-dihydropyrimidine-2(1H)-thiones with different reactants (Scheme 8) [77]. It was determined in vitro antibacterial activity of the compounds against seven bacterial strains, such as *Bacillus subtilis* MTCC 2451, *Staphylococcus aureus* MTCC 1740, *Staphylococcus epidermidis* MTCC 435, *Escherichia coli* MTCC 443, *Salmonella typhimurium* MTCC 1251, *Pseudomonas fluorescence* MTCC 103, and *Acenetobactor calcoaceticus* MTCC 127, by the agar well diffusion method, and minimal inhibition concentration (MIC) was determined. Table 1 lists the compounds with the best antibacterial activity on at least three tested strains. We note that the ethylated compounds “b” are more biologically active.

**Scheme 9:**
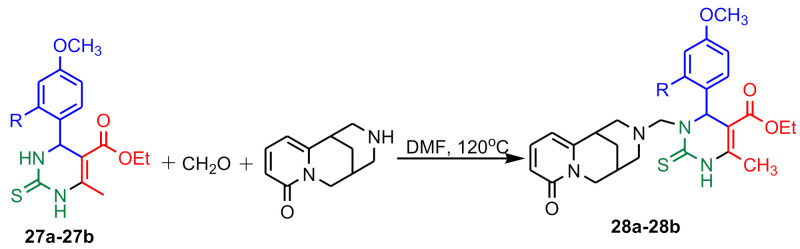
Synthesis of compounds **28a**–**28b**.

Kulakov synthesized new 3,4-dihydropyrimidin-2-thiones **28** in two steps: (i) a Biginelli reaction to obtain compounds **27** and (ii) an aminomethylation Mannich reaction to obtain 3,4-dihydropyrimidin-2-thiones grafted with alkaloid cytisine (Scheme 9) [78]. The bioscreening of **28a** revealed its pronounced antibacterial activity against the Gram-positive strains *S. aureus* and *B. subtilis*, weak activity against Gram-negative strains *P. aeruginosa* and *E. coli*, in addition to the fungus *C. albicans*.

**Scheme 10:**
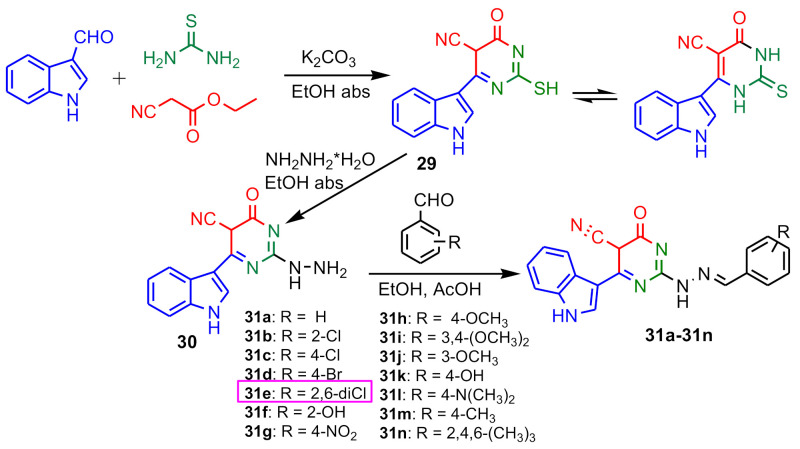
Synthesis of compounds **31a**–**31n**.

Alam et al. reported the synthesis of compounds **31a**–**31n**, using in the first step a Biginelli synthesis to obtain compound **29**, from which, by the nucleophilic attack of the hydrazine hydrate, intermediate **30** resulted. Intermediate **30** was condensed with various aldehydes, resulting in final compounds **31** (Scheme 10) [79]. The minimum inhibitory concentrations (MIC) of the compounds with antimicrobial activities are presented in Table 2. Compound **31e**, which possesses two chlorine atoms, has the best antimicrobial activity at 12.5 μg mL^−1^ against all tested strains.

**Scheme 11:**
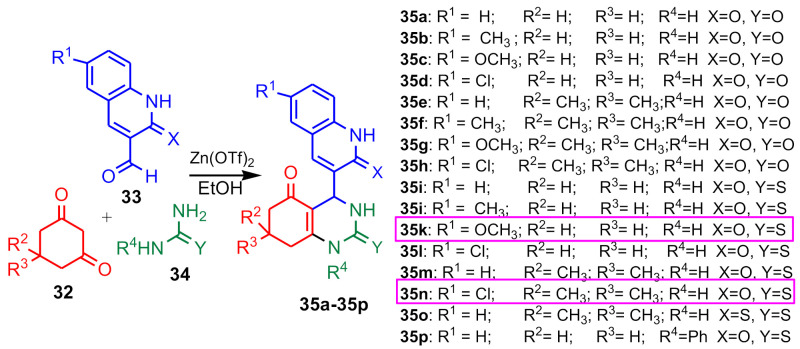
Synthesis of compounds **35a**–**35p**.

Shah and Patel synthesized new octahydroquinazolinone derivatives, using a modified Biginelli synthesis (Scheme 11) and a Lewis acid, zinc triflate, as catalyst [80]. Compounds **35a**–**35m** were screened for their antibacterial activity against *Bacillus subtilis* MTCC 441, *Clostridium tetani* MTCC 449, *Streptococcus pneumoniae* MTCC 1936, *E. coli* MTCC 443, *Salmonella typhi* MTCC 98, and *Vibrio cholerae* MTCC 3906 as well as for antifungal activity against Aspergillus fumigatus MTCC 3008 and *Candida albicans* MTCC 227. From the screening results, compound **35k** shows excellent antibacterial activity against *E. coli* (MIC = 50 μg mL^−1^) when compared with ampicillin and equivalent to chloramphenicol (MIC = 50 μg mL^−1^); compounds **35c**, **35e**, **35f**, **35g**, **35h**, **35i**, and **35l** show comparable antibacterial activity against *E. coli* (MIC = 100 μg mL^−1^) when compared with ampicillin (MIC = 100 μg mL^−1^). Compound **35n** shows excellent antibacterial activity against *S. typhi*, *S. pneumoniae*, *V. cholerae*, and *B. subtilis* (MIC = 25–50 μg mL^−1^) when compared with chloramphenicol (MIC = 50 μg mL^−1^) and ampicillin (MIC = 100 μg mL^−1^) and compounds **35o**, **35p** were found to exhibit excellent antibacterial activity against *S. typhi* when compared with ampicillin. (MIC = 100 μg mL^−1^). Antifungal screening data show that compound **35i** shows excellent antifungal activity (MIC = 100 μg mL^−1^) against *C. albicans* when compared with nystatin and griseofulvin (MIC = 100 μg mL^−1^); compounds **35a**, **35c**, **35e**–**35h**, **35j**, and **35o** show excellent antifungal activity (MIC = 500 μg mL^−1^) against *C. albicans* when compared with griseofulvin (MIC = 500 μg mL^−1^).

**Scheme 12:**
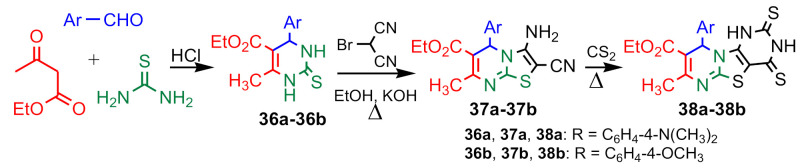
Synthesis of compounds **36**, **37**, and **38**.

Youssef and Amin synthesized new compounds **37a**–**37b** and **38a**–**38b**, using Biginelli intermediates **36a**–**36b** obtained by a classical reaction (Scheme 12) [81]. The newly heterocyclic compounds were tested for their antimicrobial activity against *Escherichia coli*, *Pseudomonas putida*, *Bacillus subtilis*, *Streptococcus lactis*, *Aspergillus niger*, *Penicillium* sp., and *Candida albican*s. All compounds showed moderate to slight inhibitory action towards the microorganisms.

**Figure 3:**
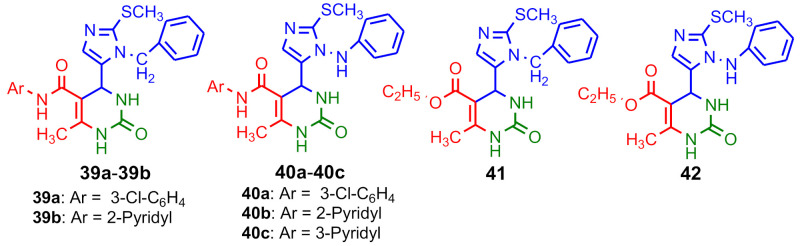
Chemical structures of compounds **39a**–**39b**, **40a**–**40c**, **41** and **42**.

Sedaghati et al. reported synthesis of new Biginelli pyrimidines **39a**–**39b**, **40a**–**40c**, **41**, and **42**, using ferric chloride as Lewis acid (Figure 3) [82]. Compounds **39a** and **42** possessed a significant antibacterial activity (MIC = 128 μg mL^−1^) against *Staphylococcus aureus* PTCC 1337 and *Pseudomonas aeruginosa* PTCC 1074, respectively. Additionally, compounds **39b**, **40a**, **40b**, **40c**, **41**, and **42** have been shown to be moderate antifungal agents against both *Candida albicans* PTCC 5027 and *Aspergillus niger* 5021 (MIC = 32–128 μg mL^−1^).

**Scheme 13:**
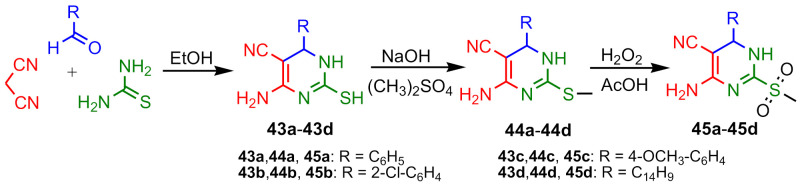
Synthesis of compounds **43a**–**43d**, **44a**–**44d**, and **45a**–**5d**.

Ghodasara et al. reported the synthesis of Biginelli compounds **43a**–**43d**, the corresponding methylated derivatives **44a**–**44d**, and their oxidized compounds **45a**–**45d** (Scheme 13) [83]. The preliminary in vitro biological activities of the compounds revealed that compounds **43a**, **44d**, **45h**, and **45j** exhibited significant (maximum) antibacterial activities against all bacterial tested strains, *S. aureus* MTCC 96, *E. coli* MTCC 443, and *B. subtilis* MTCC 441, compared with Ampicillin, Chloramphenicol, Ciprofloxacin, and Norfloxacin as standards drug, and against both fungal strains, *C. albicans* MTCC 227 and *A. niger* MTCC 282.

**Scheme 14:**
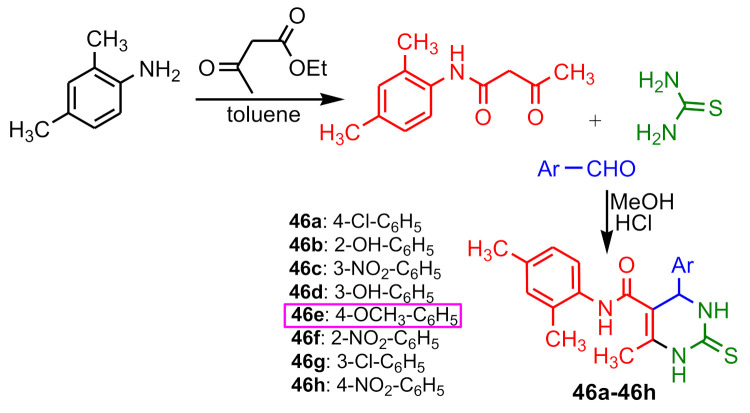
Synthesis of compounds **46a**–**46h**.

Godhani et al. reported the Biginelli synthesis of compounds **46a**–**46h** (Scheme 14) and their antimicrobial activities [84]. Compounds **46b**, **46g**, and **46h** showed good activity against *E. coli* MTCC 443 bacteria (MIC = 324.7–405.7 μM L^−1^), while compound **46e** showed excellent activity against *E. coli* (MIC = 202.93 μM L^−1^). Additionally, compounds **46b** and **46e** showed good activity against *P. aeruginosa* MTCC 1688. Compounds **46a**, **46b**, **46c**, **46d**, **46f**, **46g**, and **46h** showed moderate activity against *S. aureus* MTCC 96, with MIC = ranging from 649.4 to 811.75 μM L^−1^. Compounds **46b**, **46e**, and **46f** showed good activity against *S. pyogenus* MTCC 442 (MIC = 324.7–405.87 μM L^−1^).

**Scheme 15:**
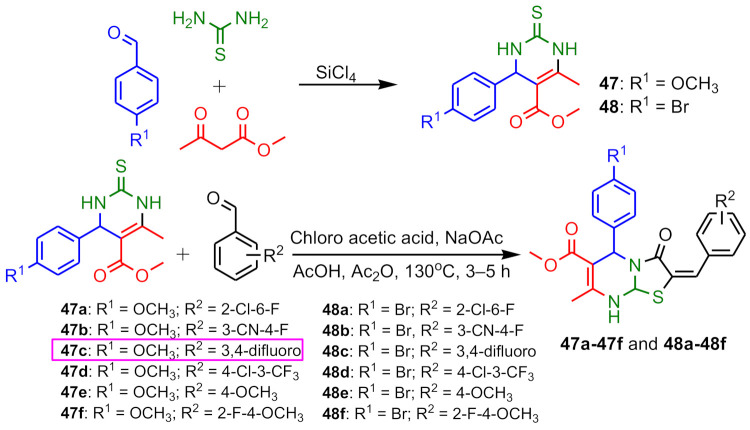
Synthesis of compounds **47a**–**47f** and **48a**–**48f**.

Umesha et al. synthesized in two steps compounds **47a**–**47f** and **48a**–**48f** (Scheme 15) [85]. Compound **48c** showed the best antimicrobial activity against all tested strains, four bacterial strains, *S. aureus*, *B. subtilis*, *S. typhi*, and *E. coli*, and two fungal strains, *A. niger* and *C. albicans*, but the other compounds also had good antimicrobial activities.

**Scheme 16:**
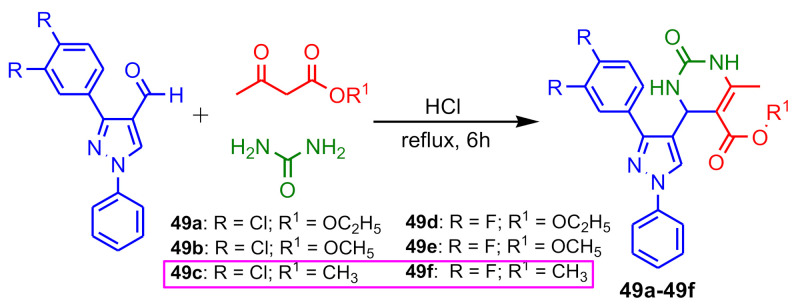
Synthesis of compounds **49a**–**49f**.

Viveka et al. synthesized compounds **49a**–**49f** by a Biginelli reaction (Scheme 16) [86]. The antibacterial screening results revealed that acetyl-substituted pyrimidinone compounds **49c** and **49f** showed a broad spectrum of antimicrobial activity against *E. coli*, *P. aeruginosa*, and *K. pneumonia* (6.25 μg mL^−1^), comparable with the standard Streptomycin (6.25 μg mL^−1^). A gradual decrease in the activity against the tested strains was noticed, with the introduction of ethoxy **49a** and **49d** and methoxy **49b** and **49e** groups, in place of acetyl substituent.

**Scheme 17:**
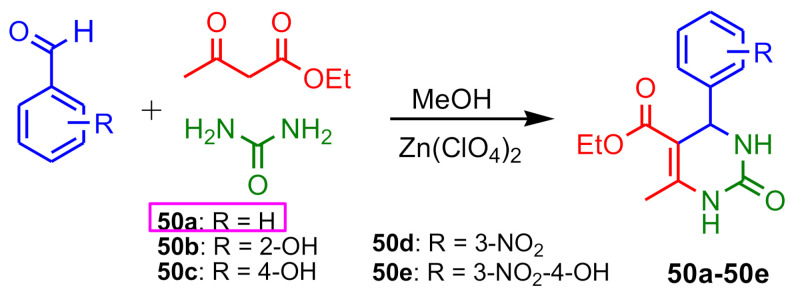
Synthesis of compounds **50a**–**50e**.

Raj et al. reported the synthesis of dihydropyrimidinones **50a**–**50e**, by a Biginelli reaction using Zn(ClO_4_)_2_ as catalyst (Scheme 17) [87]. In vitro antibacterial studies of dihydropyrimidones **50a**–**50e** were carried out against *Escherichia coli* MTCC 119, Shigella flexneri MTCC 1457, *Pseudomonas aeruginosa* MTCC 741, and *Staphylococcus aureus* MTCC 740 strains, by disk-diffusion assay. Antifungal evaluations were also carried out against two fungal strains, *Geotrichum candidum* MTCC 3993 and *Candida albicans* MTCC 227 (Table 3). From the determined MIC values, it can be said that compound **50a** had the best antimicrobial activity against all the strains tested.

**Scheme 18:**
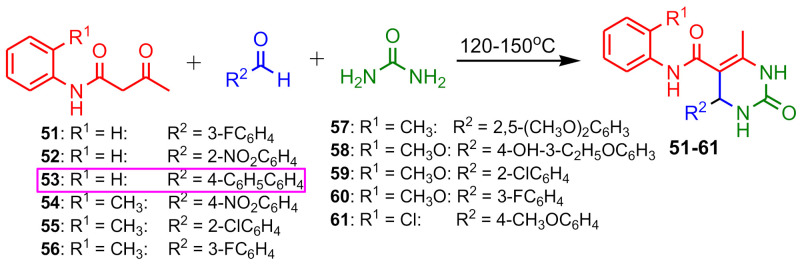
Synthesis of compounds **51**–**61**.

Gein et al. reported the synthesis of new N,6-diaryl-4-methyl-2-oxo- 1,2,3,6-tetrahydropyrimidine-5-carboxamides by a Biginelli reaction (Scheme 18) [88]. Compounds **51**–**61** showed weak antimicrobial activity against *S. aureus*, E.coli, and *C. albicans* (MIC = 250–1000 μg mL^−1^) considering dioxidine and fluconazole as standards, but good antibacterial activities for compound **53** (MIC = 250 μg mL^−1^), considering Chloramine B as standard (MIC = 250–500 mg mL^−1^).

**Figure 4:**
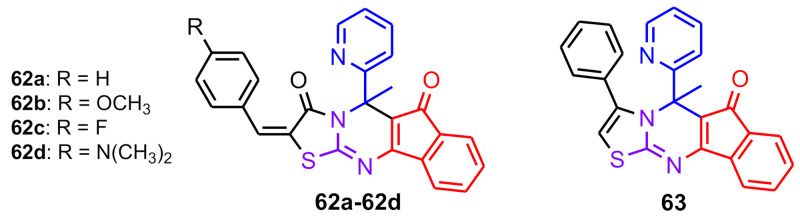
Chemical structures of compounds **62a**–**62d** and **63**.

Khalifa et al. reported the synthesis of compounds **62a**–**62d** and **63**, using a Biginelli intermediate reaction (Figure 4) [89]. Compound **62d** exhibited inhibition versus all kinds of bacterial (*Staphylococcus aureus*, *Salmonella typhimurium* and *Pseudomonas aeruginosa*) and fungal strains (*Candida albicans* and *Aspergillus flavus*). Additionally, compounds **62a**–**62c** possessed good antimicrobial activities.

Ahmad et al. reported the synthesis of some 2-amino-1,4-dihydropyrimidines by a Biginelli reaction, starting from guanidine HCl, benzaldehyde, and ethyl acetoacetate in DMF, and SnCl_2_·2H_2_O or NaHCO_3_ as catalyst, under ultrasonic irradiation [90].

**Figure 5:**
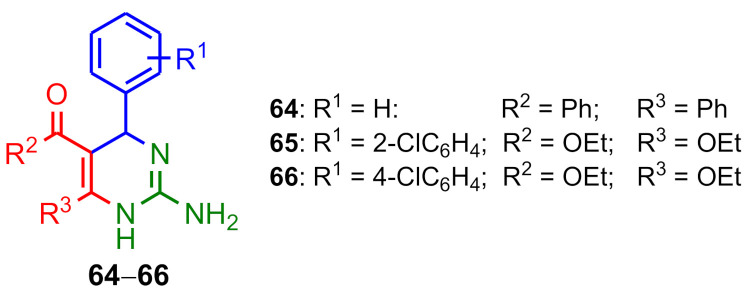
Chemical structures of compounds **64**–**66**.

The good antibacterial activities of compounds **64**, **65**, and **66** (Figure 5), against *S. aureus*, *B. subtilis*, *E. coli*, and *S. typhi*, as well as the theoretical studies, have shown that these compounds may have acceptable pharmacokinetic/drug-like properties.

**Scheme 19:**
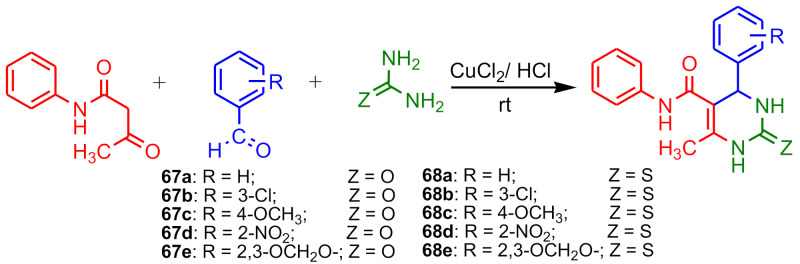
Synthesis of compounds **67a**–**67e** and **68a**–**68e**.

**Figure 6:**
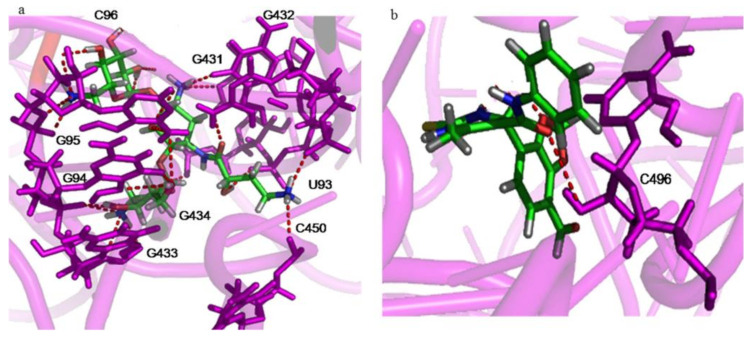
Extra precision Glide docking of human rRNA (3J3D) with (**a**) amikacin and (**b**) compound **68e**.

Ramachandran et al. reported the syntheses of dihydropyrimidinones **67** and **68** using solvent-free grindstone chemistry method catalyzed by CuCl_2_·2H_2_O and HCl (Scheme 19) [91]. All the synthesized compounds exhibited significant activity against pathogenic bacteria *Salmonella typhi* and *Staphylococcus aureus*. These dihydropyrimidinone (DHPM) derivatives also focus on the bacterial ribosomal A site RNA as a drug target. Series of docking studies were also performed for human 40S rRNA as a target. It was found that amikacin drug exhibited higher binding affinity than compound **68e**, which showed relatively low binding affinity towards human rRNA site (Figure 6).

**Scheme 20:**
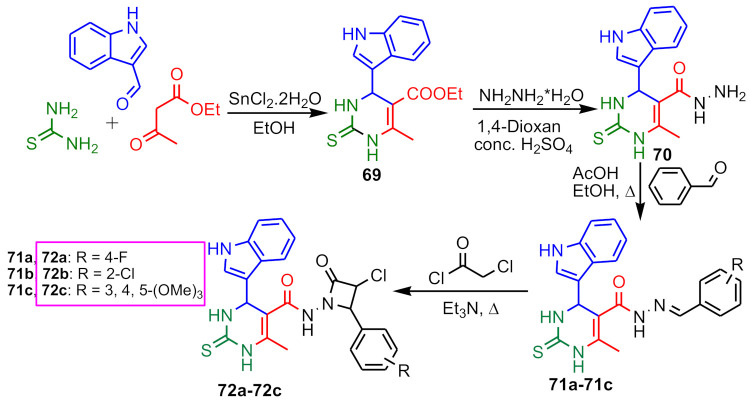
Synthesis of compounds **71a**–**71c** and **72a**–**72c**.

Desai and Bhatt reported the synthesis of compounds **72a**–**72c**, using, in the first step, a Biginelli reaction in the presence of SnCl_2_·2H_2_O catalyst to obtain compound **69**, which, in the presence of hydrazine hydrate, resulted in a hydrazide **70**, from which Schiff bases **71a**–**71c** were obtained by reaction with aromatic aldehydes. Cyclization of compounds **71** in the presence of triethylamine provided the desired β-lactams **72a**–**72c**, as shown in Scheme 20 [92]. Compounds **72a**–**72c** exhibited outstanding antimicrobial properties against almost all tested strains *S. aureus*, *S. pyogenes*, *E. coli*, *P. aeruginosa*, *C. albicans*, *A. niger*, and *A. clavatus* with MIC = 12.5–50 μg mL^−1^ for antibacterial activities and 25–100 μg mL^−1^ for antifungal activities, respectively.

**Figure 7:**
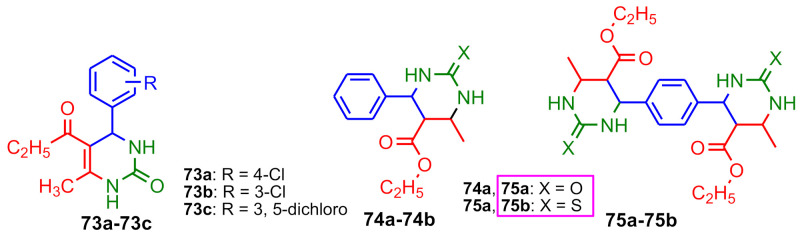
Chemical structures of compounds **73a**–**73c**, **74a**–**74b**, and **75a**–**75b**.

Attri et al. synthesized dihydropyrimidinones by a Biginelli reaction with triethylammonium acetate ionic liquid as catalyst [93]. Compounds **73a**–**73c** (Figure 6) showed the good antibacterial activity against all bacteria *Escherichia coli* MTCC 443, *Staphylococcus aureus* MTCC 3160, *Pseudomonas aeruginosa* MTCC 2581 and *Klebsiella pneumoniae* MTCC 7028, which could possibly be due to the presence of halogen atom in the molecules. Wani et al. reported the synthesis of Flucytosine analogues **74a**–**74b** and **75a**–**75b** obtained by the classic Biginelli reaction as efficient antifungal agents against *C. albicans* (Figure 7) [94]. Thus, bis-derivatives **75a**–**75b** were found to be more efficacious than their corresponding mono analogues **74a**–**74b**. Compound **75b** with two pyrimidithione rings showed high synergy with amphotericin-B and fluconazole, both followed by compounds **75a**, **74b**, and **74a**.

**Scheme 21:**
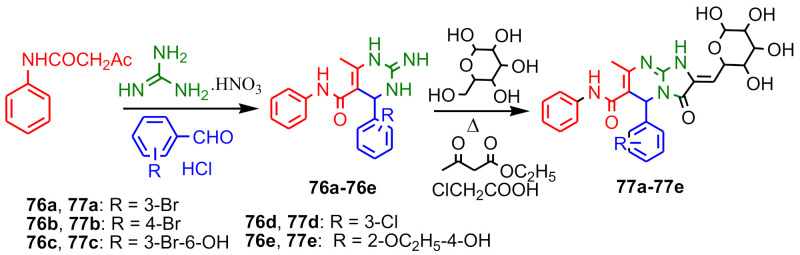
Synthesis of compounds **76a**–**76e** and **77a**–**77e**.

Rani et al. synthesized compounds **76a**–**76e** by a Biginelli reaction from 3-oxo-N-phenylbutanamide, guanidine nitrate, an aldehyde, and HCl as catalyst [95] (Scheme 21), and compounds **77a**–**77e**, from the reaction of derivatives **76** with 6-(hydroxymethyl)-tetrahydro-2H-pyran-2,3,4,5-tetraol, ethyl acetoacetate, and monochloroacetic acid. It was found that compounds **77a** and **77b** had significant activity against *S. aureus* (MIC = 2.14 × 10^−2^ μM mL^−1^), and compound **77c** was most potent against *B. subtilis* (MIC = 0.58 × 10^−2^ μM mL^−1^). Compound **77e** displayed more potent activity against *E. coli* (MIC = 1.10 × 10^−2^ μM mL^−1^), and compound **77d** was found to be most potent against *C. albicans* and *A. niger* (MIC = 1.04 × 10^−2^ μM mL^−1^).

**Figure 8:**
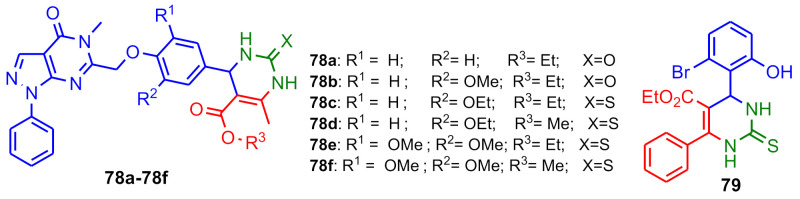
Chemical structures of compounds **78a**–**78f** and **79**.

Allam synthesized pyrazolopyrimidinone based dihydropyrimidinones **78a**–**78f** by a green Biginelli procedure catalyzed by CuCl_2_·2H_2_O (Figure 8) [96]. All compounds had good antibacterial activities against *B. subtilis*, *E. coli*, *K. pneumonia*, and *S. aureus*. Foroughifar et al. reported that tetrahydropyrimidine **79**, synthesized by a Biginelli reaction with DABCO (1,4-Diazabicyclooctane) as catalyst, had good antimicrobial activities against all tested strains *S. aureus*, *S. epidermidis*, *Bacillus cereus*, *K. pneumoniae*, *E. coli*, and *P. aeruginosa* [97].

**Scheme 22:**
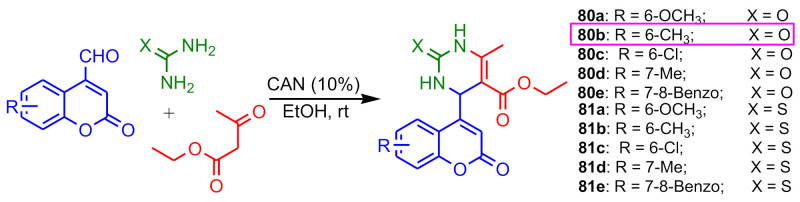
Synthesis of compounds **80a**–**80e** and **81a**–**81e**.

Naik et al. reported the synthesis of a new of 3,4-dihydropyrimidinone-coumarin analogues **80** and **81** (Scheme 22) as a new class of antibacterial agents [98]. The 3,4-dihydropyrimidinone-coumarins were evaluated for their in vitro antibacterial studies against Staphylococcus aureus, *Bacillus subtilis*, *Escherichia coli*, and *Pseudomonas aeruginosa* by Broth micro dilution method (Table 4). All compounds exhibited excellent antibacterial activity against *S. aureus* - bold values (MIC = 0.2–6.25 μg mL^−1^), but in the case of *B. subtilis*, all compounds were less active. The efficacy of substituent at C-6 position decreased in the order -CH_3_ > -7,8-Benzo > -Cl > -OCH_3_ (**b** > **e** > **c** >**a**). Similarly, compounds **81a**, **81c,** and **81e** were highly active against *E. coli*, whereas the other compounds showed significantly less activity.

**Figure 9:**
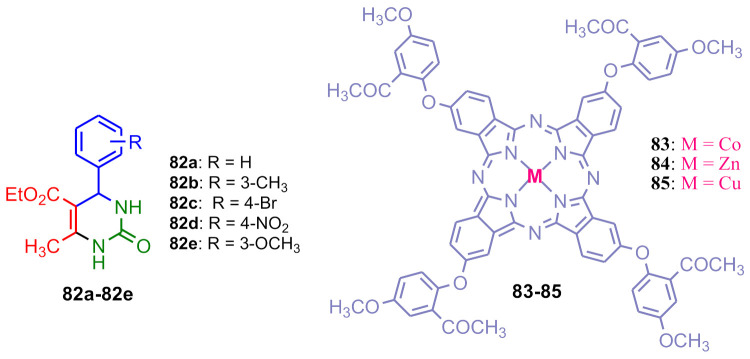
Chemical structures of compounds **82a**–**82e** and **83**–**85**.

Hamdi et al. synthesized 3,4-dihydropyrimidinones **82a**–**82e** by a modified Biginelli-type reaction with various metallophthalocyanines **83**–**85** as reusable heterogeneous as catalysts (Figure 9) [99]. The antimicrobial activity of compounds **82a**–**82e** was evaluated against *Micrococcus luteus* LB 14110, *Staphylococcus aureus* ATCC 6538, *Listeria monocytogenes* ATCC 19117, and *Salmonella typhimurium* ATCC 14028, but significant antimicrobial activity (MIC = 312 μg mL^−1^) was observed against *M. luteus*.

**Figure 10:**
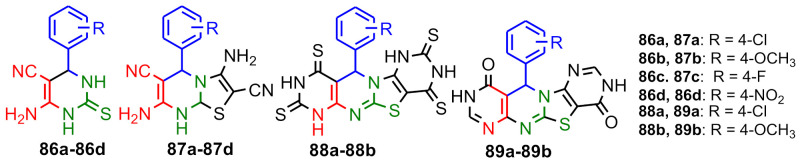
Chemical structures of compounds **86**, **87**, **88**, and **89**.

Youssef et al. synthesized 6-amino-4-aryl-2-thioxo-1,2,3,4-tetrahydropyrimidine- 5-carbonitrile derivatives **86a**–**86d** by Biginelli reaction of aromatic aldehydes, malononitrile, and thiourea in alcoholic sodium ethoxide solution [100]. The reaction of each **86** with monobromomalononitrile in ethanolic potassium hydroxide solution yielded, in each case, a single product, **87a**–**87d**. By refluxing each **87** with carbon disulphide, the corresponding compounds **88a**–**88b** were obtained. Finally, heating compounds **87** with formic acid yielded **89a**–**89b** (Figure 10). Compounds **86a**, **86b**, **87a**, **87b**, **88a**, and **89a** showed moderate to slight inhibitory action against the tested strains *Escherichia coli*, *Pseudomonas putida*, *Bacillus subtilis*, *Streptococcus lactis*, *Aspergillus niger*, *Penicillium* sp., and *Candida albicans*.

**Scheme 23:**
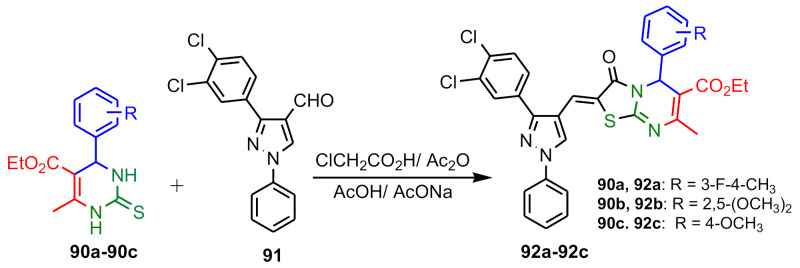
Synthesis of compounds **90a**–**90c** and **92a**–**92c**.

Viveka et al. reported the Biginelli synthesis of 3,4-dihydropyrimidinones **90a**–**90c**. Reaction of each compound **90** with pyrazole **91** gave thiazolo[2,3-b] dihydropyrimidinones **92a**–**92c** (Scheme 23) [101]. As observed in Table 5, compounds **92a**–**92c** show good antibacterial activity against all the tested species (MIC = 3.12–25 μg mL^−1^).

**Figure 11:**
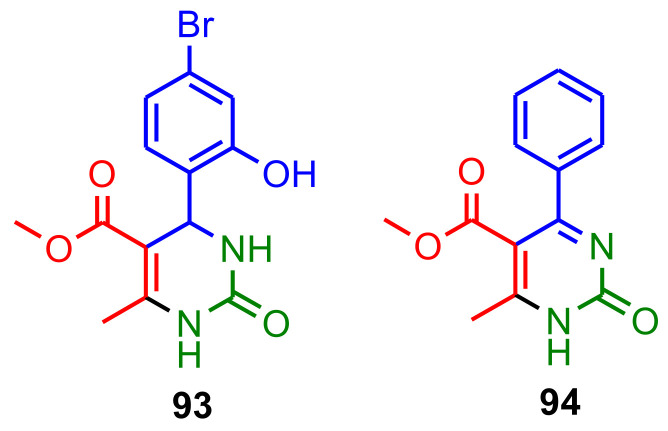
Chemical structures of compounds **93** and **94**.

Huseynzada et al. reported Biginelli synthesis catalyzed by Cu(OTf)_2_ of dihydropyrimidines, their regioselective oxidation, and their antibacterial properties [102]. Compounds **93** and **94** showed the highest inhibitory effect against *A. baumanii* and *S. aureus*, with a value of 62.5 μg mL^−1^ (Figure 11).

**Scheme 24:**
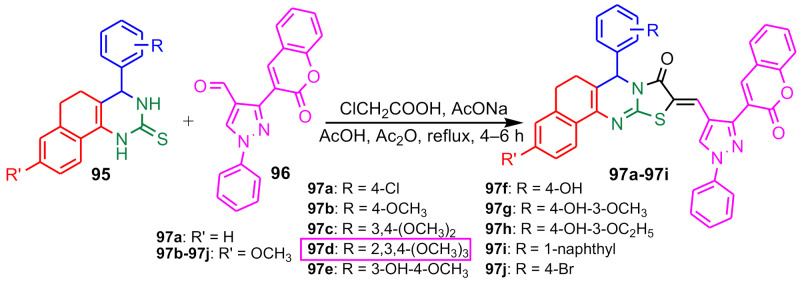
Synthesis of compounds **97a**–**97j**.

Gondru et al. reported the synthesis of a series of newly fused thiazolo[2,3-b] pyrimidinones **97** by reaction between Biginelli intermediates **95** and 3-(2-oxo-2H- chromen-3-yl)-1-aryl-1H-pyrazole-4-carbaldehyde (Scheme 24) [103] **96**. All compounds exerted significant in vitro antibacterial activity against almost all the tested bacterial strains with MICs ranging from 1.56–12.5 μg mL^−1^ (Table 6). Compound **97d** displayed inhibitory efficacies and a broader antibacterial spectrum than that of the reference drugs. Compound **97d** exhibited excellent inhibiting activity than the standard streptomycin (MIC = 6.25 μg mL^−1^) and equipotent to that of penicillin (MIC = 1.562 μg mL^−1^) against *S. aureus* and *B. subtilis* with MIC values 1.56 μg mL^−1^, being almost as active as the standard drug (MIC = 3.12 μg mL^−1^) against Gram-positive *S. epidermidis* (MIC = 3.12 μg mL^−1^). Compounds **97c** and **97e** could effectively inhibit the growth of *S. aureus* with MIC values (MIC = 1.56 and 3.12 μg mL^−1^, respectively) and *P. aeruginosa* (MIC = 6.25 μg mL^−1^). Compounds **97a**, **97b**, **97c** and **97e** have shown bioactivity against *P. aeruginosa* (MIC = 6.25 μg mL^−1^), which was better than penicillin. Compounds **97g** and **97h** showed significant activity against *S. aureus* (MIC = 3.12 μg mL^−1^).

**Scheme 25:**
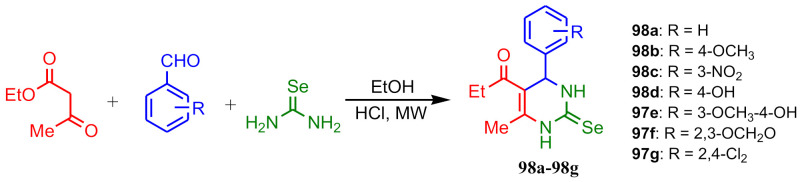
Synthesis of compounds **98a**–**98g**.

New selenoxotetrahydropyrimidines **98a**–**98g** were synthesized by Biginelli reaction of ethyl acetoacetate, substituted aromatic aldehydes and selenourea (Scheme 25) in ethanol in the presence of HCl under microwave irradiation [104]. The results of antibacterial evaluation indicated that compounds **98a** and **98e** are active against Gram-positive bacteria *Pseudomonas fluorescens*, while compounds **98b** and **98d** exhibited significant antibacterial activity against Gram-negative bacteria *Klebsellia pneumoniae* and *Escherichia coli*, respectively (Table 7). Only compound **98f** was active against both Gram-negative and Gram-positive strains, namely *Pseudomonas aeruginosa* and *Staphylococcus pyrogens*. Additionally, compounds **98c** and **98g** possessed antifungal activity against *Aspergillius janus* and *Penicillium glabrum*. In conclusion, all compounds showed inhibitory effects with a minimum inhibitory concentration of 8 μg mL^−1^.

**Scheme 26:**
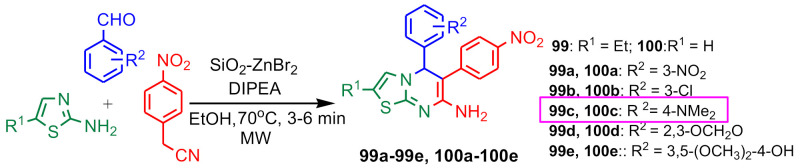
Synthesis of compounds **99a**–**99e** and **100a**–**100e**.

Devineni et al. developed a Biginelli-type reaction of substituted thiazol-2-amines, 2-(4-nitrophenyl)acetonitrile, and aromatic aldehydes, promoted by heterogeneous catalyst SiO_2_-ZnBr_2_ and diisopropylethylamine (DIPEA) as base, for the synthesis of 2,5-substituted-6-(4-nitrophenyl)-5H-thiazolo[3,2-a]pyrimidin -7-amines **99** and **100** (Scheme 26) [105]. All compounds were tested against four bacterial strains, *Staphylococcus aureus* ATCC 43300, *Bacillus subtilis* ATCC 6633, *Pseudomonas aeruginosa* ATCC 27853, and *Escherichia coli* ATCC 25922, as well as on three fungal strains *Aspergillus flavus* MTCC-1884, *Fusarium oxysporum* MTCC-1755, and *Candida albicans* ATCC 2091. Few of the synthesized compounds showed activity in the MIC range 6.25–25.0 μg mL^−1^, which was close to that of the standard drugs, tetracycline and amphotericin B (MIC = 3.125–6.25 μg mL^−1^). Compounds **99c** and **100c**, containing the dimethylamino group, showed good activity against all the tested species, in particular *B. subtilis* and *P. aeruginosa* (MIC = 6.25 μg mL^−1^).

**Figure 12:**
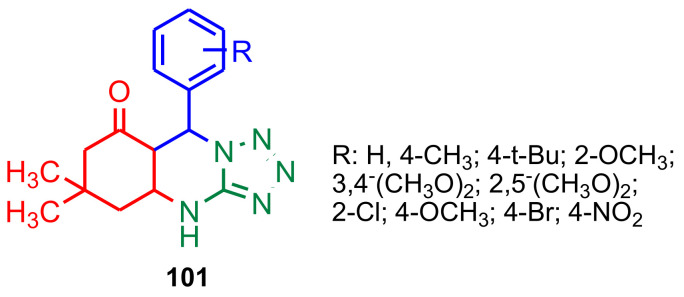
Chemical structures of compounds **101**.

Gein et al. reported that reaction of dimedone with a mixture of 5-aminotetrazole monohydrate and substituted aromatic aldehyde taken in an equimolar ratio without solvent and catalyst at a temperature of 160–170 °C for 5–10 min afforded 9-aryl-6,6-dimethyl-5,6,7,9-tetrahydrotetrazolo[5,1-b]quinazolin-8(4H)-ones **101** (Figure 12) [106]. All compounds were found to have low antibacterial and antifungal activity (MIC > 1000 μg mL^−1^).

**Scheme 27:**
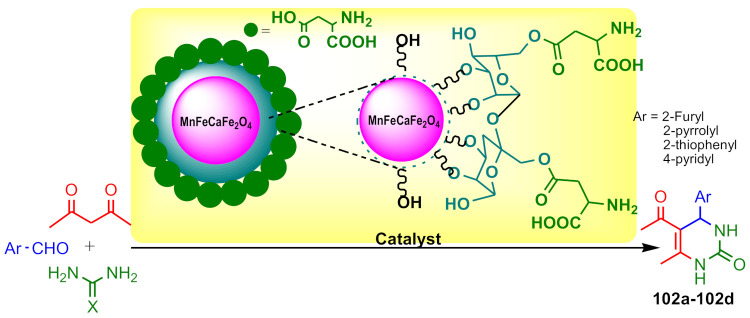
Synthesis of the compounds **102a**–**102i** catalyzed by MnFeCaFe_2_O_4_@starch@aspartic acid MNPs.

Afradi reported the synthesis of 3,4-dihydropyrimidines **102a**–**102d** using Mn_0.5_Fe_0.25_Ca_0.25_Fe_2_O_4_@starch@aspartic acid magnetic nanoparticles (MNPs) as a new nanocatalyst in a solvent free synthesis (Scheme 27) [107]. The main advantages of the Biginelli reaction in the presence of catalyst Mn_0.5_Fe_0.25_Ca_0.25_Fe_2_O_4_@starch@aspartic acid magnetic nanoparticles (MNPs) are: short reaction time, high yield, and the green and novel nature of the nanocatalyst. All synthesized compounds possessed good antibacterial activity against Gram-positive bacteria (*Staphylococcus aureus* ATCC 6538 and *Staphylococcus epidermidis* ATCC 12228) and Gram-negative bacteria (*Pseudomonas aeruginosa* ATCC 9027 and *Escherichia coli* ATCC 8739).

**Figure 13:**
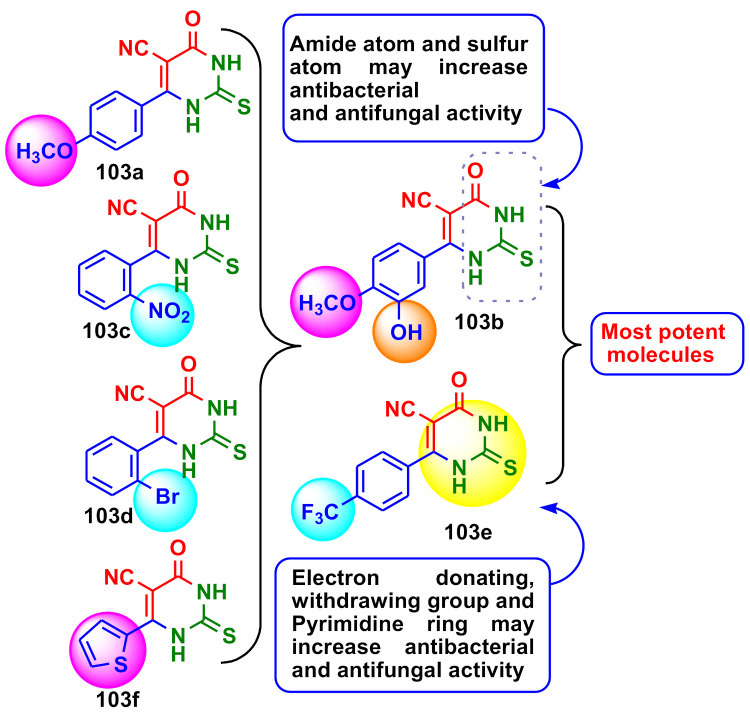
Structure−activity relationship of compounds **103a**–**103f**.

Jadhav et al. reported the diisopropyl ethyl ammonium acetate (DIPEAc)-promoted Biginelli protocol by a successive one-pot three-component reaction of aldehydes, ethylcyanoacetate/ethyl acetoacetate, and thiourea/urea to afford pharmacologically promising 1,2,3,4-tetrahydropyrimidines in high yields at room temperature [108]. All compounds were evaluated against four bacterial *Streptococcus pyogenes*, *Escherichia coli*, *Staphylococcus aureus*, *Pseudomonas aeruginosa*, and two fungal *Candida albicans*, *Aspergillus niger* strains with Ampicillin and Griseofulvin as standard drugs. Compounds **103a**, **103b**, and **103c** showed satisfactory antibacterial activity against all four bacterial pathogens due to the presence of withdrawing groups (-NO_2_ and -CF_3_) and electron-donating groups (-OH and -OCH_3_) in the molecule (Figure 13, Table 8). In addition, **103b** showed good antibacterial activity against the Gram-positive strains, *P. aeruginosa*, *E. coli*, *S. aureus*, and *S. pyogenes*, which can be significantly correlated with the presence of -OH and -OCH_3_ groups in the molecule. Additionally, compounds **103d**, **103e**, and **103f** showed potent activities against all the tested fungal strains. The best antimicrobial values are marked in bold in Table 8.

**Scheme 28:**
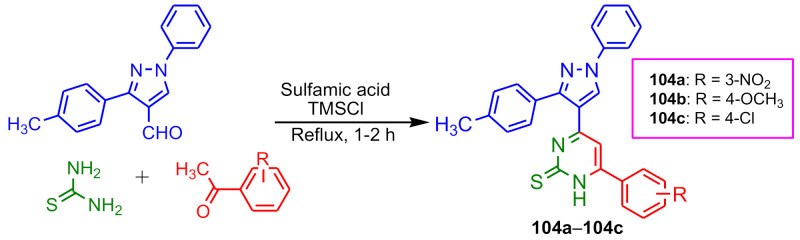
Synthesis of compounds **104a**–**104c**.

Desai et al. carried out a convenient synthesis of 20 pyrimidinthiones in the presence of thiourea and catalyst sulfamic acid (Scheme 28) [109]. Synthesized bacteria *Escherichia coli* MTCC 443, *Pseudomonas aeruginosa* MTCC 1688, *Staphylococcus aureus* MTCC 96, and *Streptococcus pyogenes* MTCC 442 and fungi *Candida albicans* MTCC 227, *Aspergillus niger* MTCC 282, and *Aspergillus clavatus* MTCC 1323 using serial dilution method. The antimicrobial activity of compounds **104a**, **104b**, and **104c** showed the best activity (of all compounds - bold values) against all tested strains (Table 9).

**Scheme 29:**
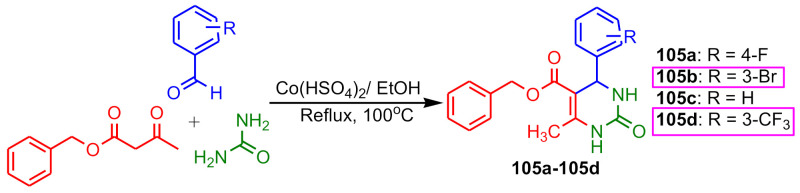
Synthesis of compounds **105a**–**105d**.

New 1,2,3,4-tetrahydropyrimidines **105** were synthesized by Biginelli reaction starting from the desirable aldehyde, benzyl 3-oxobutanoate, urea, and Co(HSO_4_)_2_ (Scheme 29) [110]. Antibacterial activity of the compounds was tested against bacterial strains *S. aureus*, *P. aeruginosa*, *E. coli* and *S. flexneri*. Compounds **105a**–**105d** showed significant growth inhibition at 40.6, 21.7, 37.8, and 19.9 μg mL^−1^ concentrations, respectively, against *S. aureus* and maximum activity at 34.0, 17.8, 101.4, and 52.4 μg mL^−1^, respectively, against *E. coli*.

**Scheme 30:**
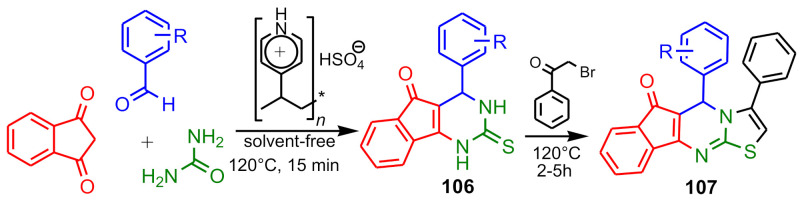
Synthesis of compounds **106** and **107**.

Rajitha et al. synthesized new 3-substituted 5-phenylindeno-thiazolopyrimidinones in two steps (Scheme 30) [111]. The first reaction is a poly(4-vinylpyridinium)hydrogen sulfate catalyzed Biginelli reaction to produce **106**, and the second is a condensation with phenacyl bromide to give **107**. Among the analogs, 4-methoxyphenyl-5-phenylindeno[1,2-d]thiazolo [3,2-a]pyrimidin-6(5H)-one showed good activity against a bacterium, Staphylococcus aureus (MIC = 25 μg mL^−1^), and a fungus, *Aspergillus niger* (zone of inhibition 20 mm).

**Scheme 31:**
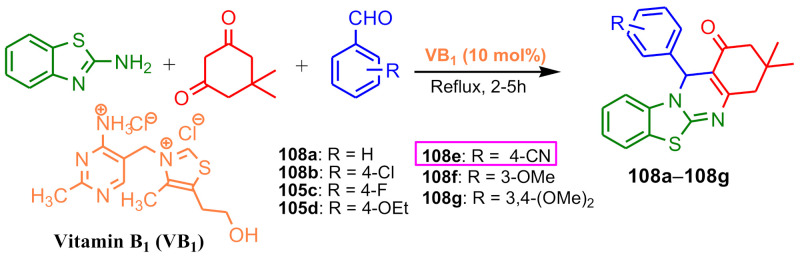
Synthesis of compounds **108a**–**108g**.

**Figure 14:**
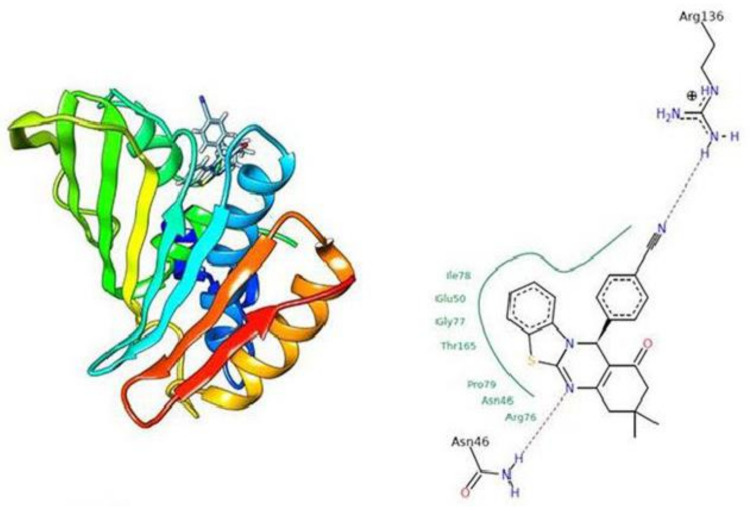
3D and 2D interaction plots of docked compound **108e** within the binding cavity of 1KZN.

Sethiya et al. reported the eco-friendly synthesis of pyrimidine derivatives **108a**–**108g** by the reaction of aromatic aldehydes, 2-aminobenzothiazole and dimedone in the presence of thiamine hydrochloride (Vitamine B_1_) as organocatalyst (Scheme 31) [112]. Molecular docking studies were performed on the synthesized compounds using *Staphylococcus aureus* dihydropteroate synthase (saDHPS) (6CLV) and DNA gyrase (1KZN) proteins. Compound **108e** was found to be the most potent and showed good binding interactions and the highest docking score against both proteins, 1KZN and 6CLV (Figure 14).

**Scheme 32:**
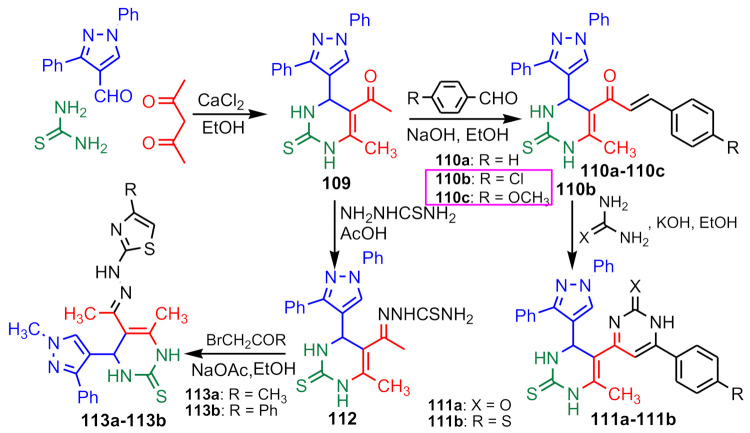
Synthesis of compounds **109**, **110a**–**110c**, **111a**, **111b**, **112**, **113a**, and **113b**.

A series of 3,4-dihydropyrimidin-2(1H)-thione compounds were synthesized from the 1-(4-(1,3-diphenyl-1H-pyrazol-4-yl)-6-methyl-2-thioxo-1,2,3,4-tetrahydro pyrimidin-5-yl)ethanone **109**, as can be seen in Scheme 32 [113]. Compounds **110b**, **111b**, **112**, and **113b** were the most potent against the tested microorganisms *Staphylococcus aureus* AUMC B.54, *Bacillus cereus* AUMC B.52, *Escherichia coli* AUMC B.53, *Pseudomonas aeruginosa* AUMC B.73, *Candida albicans* AUMC 214, and *Aspergillus flavus* AUMC 1276 (Table 10). It was stated that presence of certain electron donating groups, such as -Cl and -OCH_3_, may increase the antimicrobial activity of the compounds. These results are in line with similar results in the literature [114,115].

## 3. Biginelli Reaction Mediated Synthesis of Antitubercular Pyrimidine Derivatives

**Figure 15:**
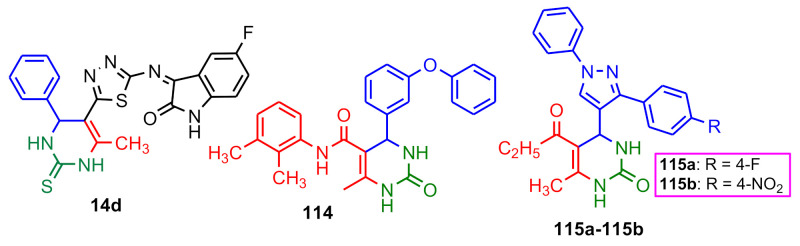
Chemical structure of compounds **14d**, **114, 115a**, and **115b**.

Tuberculosis, resulting from infection by the bacterium *Mycobacterium tuberculosis*, is a major worldwide health problem [116]. Approximately 2 million people die every year. The emergence of multi-drug resistance has forced the development of new structural classes of antitubercular agents, with several of them showing promising activity against *M. tuberculosis* [117]. Virsodia et al. synthesized new N-phenyl-6- methyl-2-oxo-4-phenyl-1,2,3,4-tetrahydro-pyrimidine-5-carboxamides using the Biginelli reaction by reacting acetoacetanilide derivatives, substituted aldehydes, and urea in methanol with a catalytic amount of HCl. Compound **114** showed 65% inhibition of *M. tuberculosis*, the best antitubercular activity (Figure 15). Additionally, 3D-QSAR studies are reported. Trivedi et al. reported the synthesis of 30 dihydropyrimidines by a classical Biginelli reaction and their in vitro antitubercular activity against *Mycobacterium tuberculosis* H37Rv [118]. Two compounds, **115a** and **115b**, with MIC of 0.02 μg mL^−1^ against *M. tuberculosis*, were found to be the most active of all and more potent than isoniazid. Akhaja et al. found that compound **14d** displayed promising antitubercular activity compared to standards Rifampicin and Izoniazid [75].

**Scheme 33:**
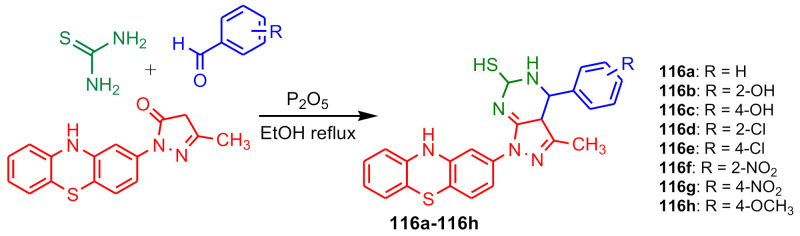
Synthesis of compounds **116a**–**116h**.

Trivedi et al. reported synthesis of phenothiazine-pyrazolo[3,4-d]pyrimidines **116a**–**116h** by Biginelli reaction in the presence of P_2_O_5_ as catalyst (Scheme 33) [119]. Compounds **116b**, **116d**, and **116f** exhibited excellent antitubercular activity with percentage inhibition of 93, 91, and 96, respectively, at a minimum inhibitory concentration (MIC) < 6.25 μg mL^−1^, whereas compounds **116a**, **116c**, **116e**, **116g**, and **116h** exhibited moderate to good antitubercular activity, with a percentage inhibition of 75, 68, 74, 54, and 63, respectively, at MIC > 6.25 μg mL^−1^.

**Figure 16:**
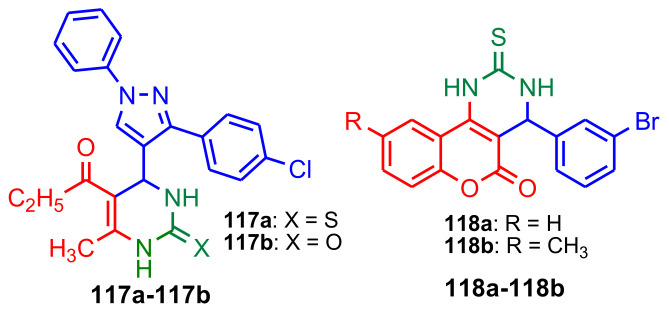
Chemical structure of compounds **117a**–**117b**, **118a**–**118b**.

Yadlapalli implemeted a Biginelli reaction for the synthesis of 4-aryl-3,4-dihydro-2(1H)-pyrimidone esters possessing lipophilic carbamoyl groups [120]. Compounds **117a** and **117b**, with a MIC value of 0.125 and 0.25 μg mL^−1^, were found to be the most potent in the series (Figure 16). Ambre et al. reported the synthesis of 16 compounds, 4-(substituted) phenyl-2-thioxo-3,4-dihydro-1H- chromino[4,3-d]pyrimidin-5-one and 4-(substituted) phenyl-3,4-dihydro-1H- chromino[4,3-d]pyrimidine-2,5-dione analogs as antitubercular agents by a classical. Biginelli reaction between a substituted aldehyde, 6-substituted-4-hydroxy coumarin, urea (or thiourea), and p-toluenesulfonic acid as catalyst. Compounds **118a** and **118b**, with MIC of 59% and 61%, respectively, were found to be the most potent in these series [121].

**Scheme 34:**
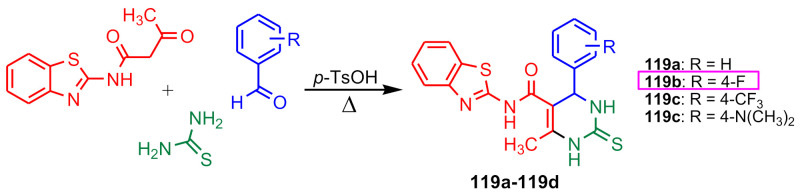
Synthesis of compounds **119a**–**119d**.

Chikhale et al. synthesized a series of N-(benzo[d]thiazol-2-yl)-6-methyl -4-phenyl-2-thioxo-1,2,3,4-tetrahydropyrimidine -5-carboxamide by a Biginelli reaction (Scheme 34) [122]. It was found that compounds **119a**–**119d** exhibited an MIC between 0.08 and 0.09 μM, which is found to be better than the standard reference Isoniazid with MIC of 0.2 μM. The very good antitubercular activity was correlated with the presence of the fluorine atom in the molecule.

## 4. Antioxidant Activity of Antimicrobial Pyrimidine Derivatives

**Figure 17:**
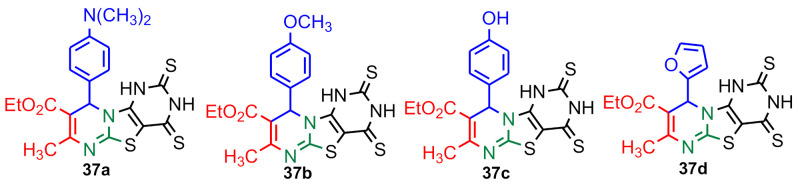
Chemical structure of compounds **37a**–**37d**.

Youseff et al. found that compounds **37a** and **37b** showed considerable inhibitory activity in the hemolysis assay (Table 11) [81]. Compounds **37c** and **37d** (Figure 17) showed moderate antioxidant and inhibitory activity (Table 12). Additionally, the antioxidant assay by ABTS method showed that compounds **37a**, 3**7b**, **37c**, and **37d** showed potent antioxidant activity.

**Figure 18:**
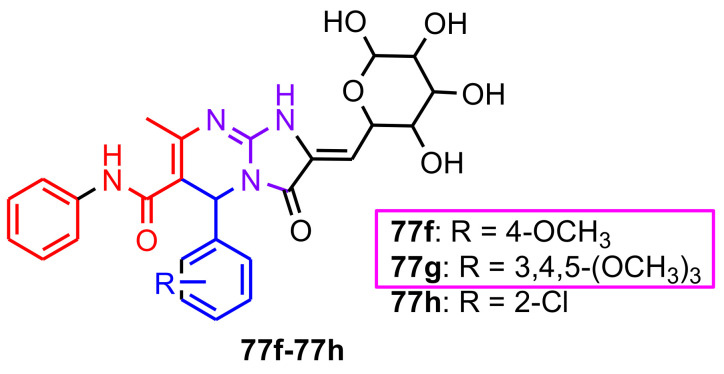
Chemical structures of compounds **77f**–**77h**.

Viveka et al. reported compounds **49c** (89.41%) and **49f** (83.34%) exhibited excellent DPPH radical scavenging activity, as compared to glutathione (89.09%) [86]. Attri et al. reported that that compounds **73a**–**73c** possess good-to-moderate antioxidant activity in comparison to the standard gallic acid and quercetin [93]. Rani et al. reported that compounds **77f** and **77g** exhibited excellent in vitro antioxidant activity due to the presence of electron releasing groups on benzylidene portion (Figure 18) [95].

## 5. Anticancer Activity of Antimicrobial Pyrimidine Derivatives

Yadlapalli reported that compound **117a**, with excellent antitubercular activity against MTB H37Rv, showed moderate anticancer activity against MCF-7 breast cancer cell lines [120]. Compound **77h** was found to be the most potent anticancer agent (IC_50_ = 57.65 mg mL^−1^) of all compounds **77** at a dose of 10^−4^ M against human breast (MCF-7) cancer cell line and was comparable with Adriamycin as the standard [95]. Gondru et al. showed that derivatives **97a**, **97g**, **97h**, and **97j** have shown moderate antiproliferative potency against the HepG2 tumor cell line with an average percentage of inhibition, ranging from 39.09 to 40.35 at cell lines [103].

## 6. Anti-Inflammatory Activity of Antimicrobial Pyrimidine Derivatives

Gelatinases are present in the physiologic system and play a key role in inflammation and autoimmunity states. Activated inflammatory cells and dermal fibroblasts can express several proteinases designated as matrix metalloproteinases (MMPs) able to degrade all connective tissue macromolecules [98]. Among these are gelatinases, e.g., MMP-2 and MMP-9, which, together with interstitial collagenase, have been assumed to be of importance in connective tissue remodeling after inflammation. The obtained results revealed that all compounds **80a**–**80e** and **81a**–**81e** were highly active against MMP-2 (72 kDa gelatinase A). Similarly, the compounds **80e**, **81a**, **81b**, **81d**, and **81e** were highly active against MMP-9 (92 kDa gelatinase B), whereas the compounds **80b** and **80d** showed slight inhibitory activity, and rest of the compounds were not active against MMP-9 (Table 13).

**Figure 19:**
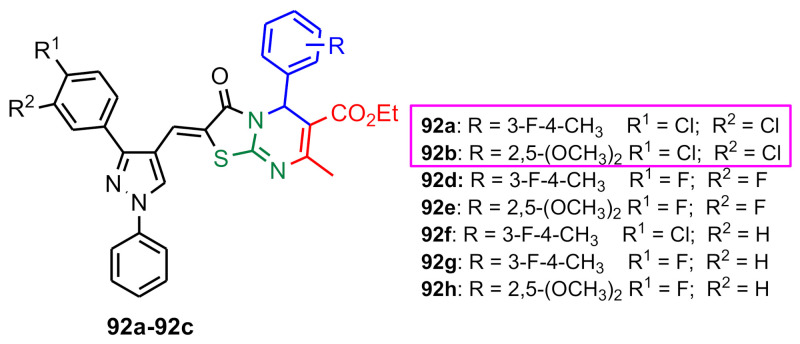
Chemical structures of compounds **92a**, **92b**, **92e**–**92h**.

Alam et al. screened compounds **31a**–**31n** for their anti-inflammatory activity using Winter et al. method. The results showed inhibition of edema ranging from 32.72% to 71.14%. The compounds **31c**, **31d**, and **31e** (Scheme 10) showed 65.45%, 67.07%, and 71.14%, respectively, inhibition of edema [79]. From the anti-inflammatory activity result analysis, Viveka et al. observed that compounds **92a**, **92b**, **92d**, **92e**, **92f**, **92g**, and **92h** showed good activity, with 67.61 to 85.33% inhibition of the edema (Figure 19). The compounds **92a** (85.33), **92d** (81.32), and **92h** (80.75) showed potent anti-inflammatory activity compared with the other test compounds and are comparable with the standard, indomethacin (86.76). This emphasizes the presence of the 3F-4CH_3_- substituted phenyl ring on the 5th position of the 3-oxothiazolopyrimidine nucleus in this pyrazole series [101]. Additionally, El-Emary et al. found that compounds **110b** and **112b** (Scheme 29) had the most anti-inflammatory activity, comparable to that of Indomethacin [113].

## 7. Analgesic Activity of Antimicrobial Pyrimidine Derivatives

**Figure 20:**
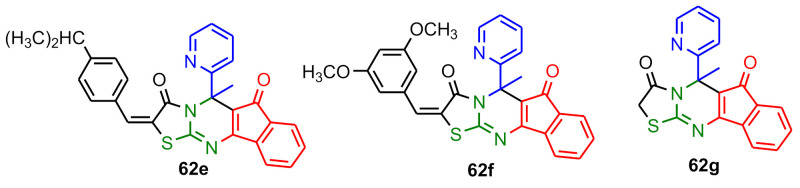
Chemical structures of compounds **62e**, **62f**, and **62g**.

Alam et al. reported that compounds **31c**, **31d**, and **31e** with analgesic activity (expressed as % protection) of 44.35%, 47.01%, and 50.36%, respectively, have analgesic properties, considering Indomethacin as the standard (60.30%) [79]. Khalifa et al. reported the descending order of the central analgesic potencies of compounds **62e**–**62g** after 90 min, as compared to Tramadol, which were 97.2, 97.2, 89.0, and 78.0% for compounds **62e**, **62f**, **62g**, and **62b**, respectively (Figure 4 and Figure 20) [84]. Gein et al. reported that all studied compounds **51**–**61** (Scheme 16) were found to have high analgesic activity, as compared with sodium Metamizole (47.7%) or Nimesulide (75.5%) with analgesic activity between 60.8% and 84.0% [88].

## 8. Antiviral Activity of Antimicrobial Pyrimidine Derivatives

**Figure 21:**
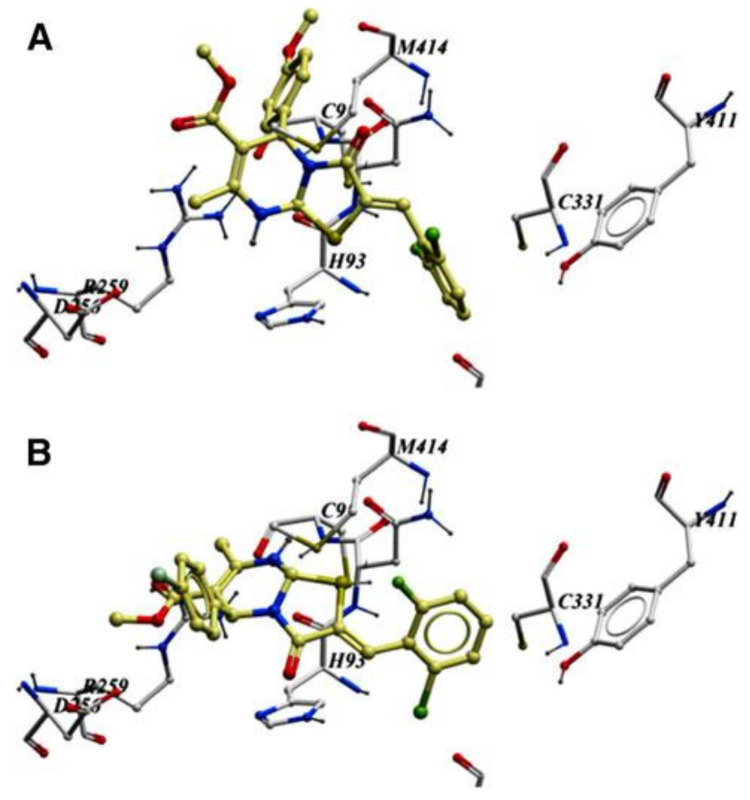
Intermolecular interactions with of the compounds **47a** (**A**) and **48a** (**B**) (Scheme 13) at the active site of human inosine monophosphate dehydrogenase (IMPDH) type II (PDB ID: 1NFB), where Cys331 is the catalytic residue. Both compounds exhibited imperative hydrogen bonding with a number of explicit residues of the active site of IMPDH.

Umesha et al. reported the antiviral studies for the selected compounds, in which **48a** exhibited 73.69% and 54.42% of inhibition of buffalopox and camelpox viruses, respectively (Scheme 15) [85]. The calculated docking (ΔE) and binding (ΔG) energies of **47a** were −54.2 and −8.7 kcal Mol^−1^, respectively, whereas for compound **48a**, the calculated energy values were −58.6 and −10.4 kcal Mol^−1^, respectively. An O4 oxygen atom of compound **47a** exhibited long-distance (3.24 Å) hydrogen-bond interactions with the N atom of the residue Met414 of human IMPDH, and most of the atoms of the molecule showed hydrophobic interactions with Cys331, Met70, His93, Asn94, and Cys95 (Figure 21A). Compound **4a** exhibited at least three different long-distance hydrogen-bond interactions. An O1 atom showed with the NH_2_ atom of Arg259 with a distance of 3.06 Å, an O3 atom showed with the NE2 atom of His93 with a distance of 2.87 Å, and a Cl atom showed with the O atom of Pro69 with a distance of 2.87 Å (Figure 21B). Additionally, other atoms of the molecule showed several important hydrophobic interactions against Asp256, Asn94, Met414, Asp71, and Tyr411 with different distances. These results showed that the relatively higher antiviral activity of compound **48a** than **47a** may due to lower docking and binding energies, as well as higher hydrogen and hydrophobic interactions.

**Figure 22:**
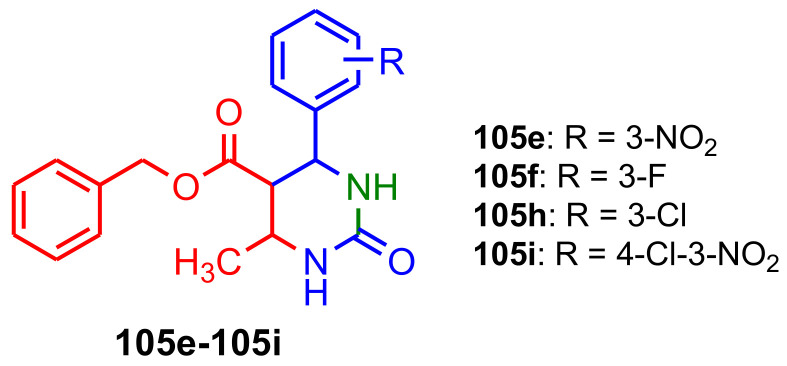
Chemical structures of compounds **105e**, **105f**, **105h** and **105i**.

Razzaghi-Asl et al. reported that trifluoromethyl group at the meta-position of phenyl ring provided a potent anti-HIV-1 property to the compound **105d** (Scheme 29) [110]. Similarly, compounds bearing nitro **105e**, fluorine **105f**, bromine **105b**, and chlorine **105h** groups at the meta-position as well as chlorine and nitro groups at para- and meta-positions **105i** (Figure 22) of phenyl were less potent, with an inhibition rate of P24 expression (%) in 100 μM of 52.25, 10.67, 12.93, 50.21, and 61.43, respectively. The presence of fluorine at meta position showed better anti-HIV-1 activity than the para-position **105d** (10.67 vs. 4.75% in 100 μM).

## 9. Antiparasitic Activity of Antimicrobial Pyrimidine Derivatives

**Figure 23:**
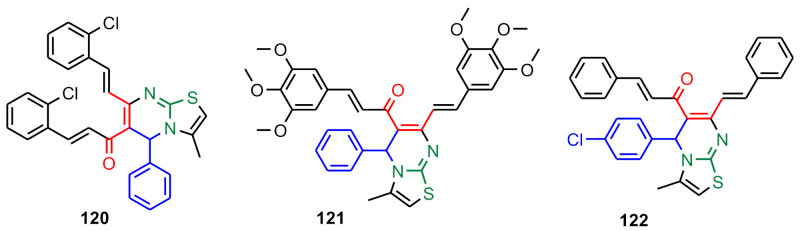
Chemical structures of compounds **120**, **121**, and **122**.

Rajanarendar reported that compounds **6b** and **6d** are proved to be lethal for mosquito larvae, with LC_50_ concentration, representing the concentration in ppm that killed 50%, of 0.85 and 0.88, respectively (Scheme 4) [72]. Thus, pyrimidine compounds **6b** and **6d** can be useful as more toxic substances to kill mosquito larvae. Fatima et al. reported the synthesis of three Biginelli compounds **120**, **121**, and **122** more potent than the standard drug Chloroquine against K1 strains of P. falciparum, with an IC_50_ (μg mL^−1^) of 0.56, 0.5 and 0.5, respectively (Figure 23) [123]. These compounds with three different pharmacophores have potential to be exploited in medicinal chemistry.

## 10. Conclusions

This review summarizes the recent Biginelli syntheses of pyrimidine compounds with antimicrobial properties, as well as their biological activities mentioned in the literature. Regarding the Biginelli synthesis of pyrimidine compounds, the presentation clearly shows that the catalyst has an important role in the development of the reaction and in obtaining a high yield. Derivatization of Biginelli compounds leads in most cases to compounds with stronger antimicrobial properties than the initial Biginelli dihydropyrimidines. Additionally, the presence of another heterocycle in the final molecules, such as pyrazole, thiazole, isoxazole, imidazole, benzothiazole, phenothiazine, 1,3,4-thiadiazole, coumarin, chromene, indole, and quinoline, potentiate the antimyrobial activity of pyrimidine compounds. In general, thiopyrimidine compounds have a stronger antimicrobial activity than pyrimidinone compounds. Selenopyrimidine compounds generally have better antimicrobial activity than pyrimidinone. Additionally, the presence of certain groups grafted on the benzimidazole and pyrazole nuclei, such as -NO_2_, -CN, -F, -CF_3_, -CN, -COOCH_3_, -NHCO, -CHO, Cl, -OH, OCH_3_, OC_2_H_5_, and -N(CH_3_)_2_, increases the antimicrobial activity of the compounds [124,125,126,127]. However, there are quite a few studies performed on the structure–properties relationship for these compounds, as well as few studies of molecular mechanics, DFT, through which to achieve the directed synthesis of some biologically active molecules. Therefore, we hope that this article will be a starting point for conducting new theoretical studies and syntheses of new compounds for the synthesis of improved antimicrobial compounds that possess other biological activities.

## Figures and Tables

**Table 1:** Minimum inhibitory concentration of compounds **17**–**23**.

Compound	Minimum Inhibitory Concentration MIC (μg mL^−1^)						
	PF	ST	SA	EC	BS	SE	AC
**17b**	15	-	12	-	-	-	55
**20a**	-	27	54	1	2	2	-
**20b**	14	1	6	17	21	13	36
**23b**	14	28	-	-	-	46	-
**25b**	-	13	26	54	50	20	-

PF: *P. fluorescence*; ST: *S. typhimurium*; SA: *S. aureus*; EC: *E. coli*; BS: *B. subtilis*; SE: *S. epidermidis*; AC: *A. calcoaceticus*.

**Table 2:** Minimum inhibitory concentration MIC (μg mL^−1^) of compounds **31**.

Compounds	*Staphylococcus aureus*	*Escherichia coli*	*Rhizopus oryza*	*Penicillium citrum*
**31b**	50	>100	50	>100
**31c**	50	50	50	>100
**31d**	50	50	25	25
**31e**	12.5	12.5	12.5	12.5
**31i**	25	25	50	50
Norfloxacin	6.25	6.25	-	-
Fluconazole	-	-	6.25	6.25

**Table 3:** In vitro antibacterial activities of compounds **50a**–**50e**.

Compounds	MIC (μg mL^−1^)					
	Sa	Pa	Sf	Ec	Gc	Ca
**50a**	15 ± 1	30 ± 0.5	71	72 ± 2	40 ± 1	99 ± 1
**50b**	15	>100	>100	>100	60 ± 3	>100
**50c**	20 ± 3	18 ± 1	73	75 ± 2	>100	99 ± 1
**50d**	80 ± 4	82 ± 2	>100	90 ± 4	>100	>100
**50e**	90 ± 2	91 ± 2	>100	85 ± 4	>100	>100

Sa: *S. aureus* MTCC 740; Pa: *P. aeruginosa* MTCC 741; Sf: *S. flexneri* MTCC 1457; Ec: *E. coli* MTCC 119; Gc: *G. candidum* MTCC 3993; CA: *C. albicans* MTCC 227.

**Table 4:** In vitro antibacterial activity (MIC) of compounds **80a**–**80e** and **81a**–**81e**.

Compound	Minimum Inhibitory Concentrations (MIC) (μg mL^−1^)			
	*S. aureus*	*B. subtilis*	*E. coli*	*P. aeruginosa*
**80a**	**0.4**	12.5	50	100
**80b**	**0.8**	100	50	25
**80c**	3.12	100	25	50
**80d**	**0.2**	50	12.5	100
**80e**	**0.4**	100	25	25
**81a**	**0.4**	100	**1.6**	100
**81b**	**0.8**	50	25	100
**81c**	**0.8**	50	**0.4**	100
**81d**	6.25	50	25	100
**81e**	**0.4**	100	**0.4**	100
Ciprofloxacin	2	2	2	<4

**Table 5:** In vitro antibacterial activity (MIC) of compounds **92a**–**92c**.

Compound	Minimum Inhibitory Concentrations (MIC) (μg mL^−1^)			
	*E. coli*	*S. aureus*	*P. aeruginosa*	*K. pneumoniae*
**92a**	3.12	3.12	12.5	6.25
**92b**	3.12	6.25	12.5	3.12
**92c**	6.25	25	6.25	6.25
Streptomycin	2.8	2.5	3.8	2.8

**Table 6:** In vitro antibacterial activity (MIC) of compounds **97a**–**97i**.

Compounds	Minimum Inhibitory Concentrations (MIC) (μg mL^−1^)					
	*S. aureus*	*B. subtilis*	*S. epidermitis*	*E. coli*	*K. pneumoniae*	*P. aeruginosa*
**97a**	-	-	50 ± 0.22	-	50 ± 0.63	6.25 ± 0.21
**97b**	25 ± 0.62	25 ± 0.46	12.5 ± 0.90	50 ± 0.32	50 ± 0.79	6.25 ± 0.79
**97c**	1.56 ± 0.22	12.5 ± 0.71	12.5 ± 0.3	12.5 ± 0.44	12.5 ± 0.58	6.25 ± 0.15
**97d**	1.56 ± 0.35	1.56 ± 0.45	3.12 ± 0.66	6.25 ± 0.70	6.25 ± 0.40	12.5 ± 0.23
**97e**	3.12 ± 0.28	6.25 ± 0.19	12.5 ± 0.37	50 ± 0.68	6.25 ± 0.30	6.25 ± 0.16
**97f**	6.25 ± 0.49	6.25 ± 0.33	12.5 ± 0.38	50 ± 0.42	12.5 ± 0.22	12.5 ± 0.50
**97g**	3.12 ± 0.28	12.5 ± 0.42	12.5 ± 0.31	25 ± 0.20	50 ± 0.40	12.5 ± 0.36
**97h**	3.12 ± 0.19	6.25 ± 0.20	12.5 ± 0.45	6.25 ± 0.43	12.5 ± 0.22	6.25 ± 0.38
**97i**	-	-	-	12.5 ± 0.25	-	25 ± 0.55
Streptomycin	6.25 ± 0.25	6.25 ± 0.70	3.125 ± 0.45	6.25 ± 0.82	3.125 ± 0.96	1.562 ± 0.69
Penicillin	1.56 ± 0.21	1.562 ± 0.65	3.125 ± 0.22	12.5 ± 0.35	6.25 ± 0.88	12.5 ± 0.74

**Table 7:** In vitro antibacterial activity (MIC) (μg mL^−1^) of compounds **98a**–**98g**.

Comp.	Gram-Positive Bacteria				Gram Negative Bacteria				Fungi			
	Ec	Kp	Pa	Pf	Sa	Bs	Sp	Aj	Pg	An	Fo	As
**98a**	32	16	32	**8**	32	32	16	32	32	32	16	16
**98b**	32	**8**	32	16	16	32	32	32	16	32	32	16
**98c**	16	32	32	32	32	16	16	**8**	16	16	16	16
**98d**	**8**	32	16	32	32	16	32	32	16	3	32	32
**98e**	32	32	32	**8**	32	32	64	16	16	16	16	16
**98f**	16	16	**8**	32	16	16	8	32	16	32	32	32
**98g**	32	32	16	16	32	32	32	16	**8**	16	32	16
Amoxicilin	4	4	4	4	2	2	4	2	-	-	-	-
Fluconazole	-	-	-	-	-	-	-	-	*2*	*2*	*2*	*2*

Ec: *Escherichia coli*; Kp: *Klebsiella pneumoniae*; Pa: *Pseudomonas aeruginosa*; Pf: *Pseudomonas fluorescens*; Sa: *Staphylococcus aureus*; Bs: *Bacillus subtilis*; Sp: *Staphylococcus pyrogens*; Aj: *Aspergillus janus*; Pg: *Penicillium glabrum*; An: *Aspergillus niger*; Fo: *Fusarium oxysporum*; As: *Aspergillus sclerotioum*.

**Table 8:** In vitro antibacterial activity (MIC) (μg mL^−1^) of compounds **103a**–**103f**.

Compounds	Minimum Inhibitory Concentrations (MIC) (μg mL^−1^)					
	*E. coli*	*P. aeruginosa*	*S. aueus*	*S. pyogenes*	*C. albicans*	*A. niger*
**103a**	**50**	**25**	500	**50**	500	250
**103b**	**50**	**12.5**	**25**	250	100	250
**103c**	**12.5**	**25**	100	**50**	500	250
**103d**	100	500	100	100	100	**25**
**103e**	500	500	500	250	**12.5**	**50**
**103f**	100	100	250	**50**	50	100
Ampicillin	50	50	200	200	-	-
Griseofulvin	-	-	-	-	500	100

**Table 9:** In vitro antibacterial activity (MIC) (μg mL^−1^) of compounds **104a**–**104c**.

Compound	Bacteria				Fungi		
	Ec	Pa	Sa	Sp	Ca	An	Ac
**104a**	**12.5**	250	250	250	1000	250	250
**104b**	100	200	500	200	**12.5**	**50**	500
**104c**	100	250	**12.5**	**12.5**	500	1000	1000
Ciprofloxacin	25	25	50	50	-	-	-
Nystatin	-	-	-	-	100	100	100

Ec: *Escherichia coli*; Pa: *Pseudomonas aeruginosa*; Sa: *Staphylococcus aureus*; Sp: *Streptococcus pyogenes*; Ca: *Candida albicans*; An: *Aspergillus niger*; Ac: *Aspergillus clavatus*.

**Table 10:** In vitro antibacterial activity (MIC) (μg mL^−1^) of compounds **109**–**113**.

Compounds	Diameter of Zone of Inhibition (mm)/% Inhibition with Reference to Standard					
	*S. aueus*	*B. cereus*	*E. coli*	*P. aeruginosa*	*C. albicans*	*A. flavus*
**109**	15 (46)	19 (49)	22 (81)	14 (82)	-	-
**110a**	9 (50)	-	-	-	-	-
**110b**	13 (72)	16 (66)	31 (>100)	23 (>100)	11 (39)	27 (64)
**110c**	10 (55)	13 (54)	15 (56)	-	-	-
**111a**	12 (66)	9 (37)	13 (48)	-	-	21 (50)
**111b**	19 (>100)	29 (>100)	33 (>100)	26 (>100)	10 (36)	36 (86)
**112**	11 (61)	35 (>100)	11 (41)	8 (47)	-	-
**113a**	12 (66)	10 (42)	13 (48)	8 (47)	19 (68)	26 (62)
**113b**	22 (>100)	29 (>100)	37 (>100)	24 (>100)	-	44 (>100)
Chloramphenicol	18	24	27	17	-	-
Clotrimazole	-	-	-	-	28	42

**Table 11:** Antioxidant assays by erythrocyte hemolysis (A/B).

Compound	Absorbance of Sample (A)	Hemolysis (%)
Complete hemolysis with distilled water (B)	0.660	-
**37a**	0.035	5.22
**37b**	0.031	4.68
**37c**	0.045	6.92
**37d**	0.051	8.02

**Table 12:** Antioxidant assays by ABTS method (Abs. (control) − Abs.(test)/Abs.(control) × 100).

Compound	Absorbance of Sample (A)	Inhibition (%)
ABTS control	0.54	0
Ascorbic acid	0.06	88.8
**37a**	0.10	81.5
**37b**	0.12	77.7
**37c**	0.15	72.2
**37d**	0.13	75.9

**Table 13:** The in vitro anti-inflammatory results of compounds **80a**−**80e** and **81a**−**81e** with % bands and % inhibition of MMP-2 and MMP-9.

Compound	% Bands of MMP		% Inhibition of MMP	
	MMP-9	MMP-2	MMP-9	MMP-2
**80a**	20	10	**80**	90
**80b**	15	05	**85**	95
**80c**	100	15	-	85
**80d**	30	05	70	95
**80e**	10	04	**90**	96
**81a**	100	18	-	82
**81b**	90	13	10	87
**81c**	100	15	-	85
**81d**	70	20	30	80
**81e**	05	10	**95**	90
Tetracycline	00	00	100	100

## Data Availability

Not available.
